# Prognosis in Parkinson's Disease: An Individual Patient Data Meta‐Analysis of Six European Incidence Cohorts

**DOI:** 10.1002/mds.70303

**Published:** 2026-04-11

**Authors:** Angus D. Macleod, David J. McLernon, Marta Camacho, Caroline H. Williams‐Gray, Rachael A. Lawson, Alison J. Yarnall, David Bäckström, Lars Forsgren, Jodi Maple‐Grødem, Guido Alves, Ole‐Bjørn Tysnes, Carl E. Counsell, Parkinson's Incidence Cohorts Collaboration, Thomas Foltynie, Sarah L. Mason, Ruwani Wijeyekoon, Jonathan Evans, David P. Breen, Gemma Cummins, Krista Farrell, Kirsten Scott, Tom Stoker, Julia Greenland, Natalie Valle Guzman, Lucy Collins, Simon Stott, Jan Linder, Mona Edström, Jörgen Andersson, Linda Eriksson, Gun‐Marie Hariz, Magdalena Domellöf, Michaela Dreetz Gjerstad, Kenn Freddy Pedersen, Elin Bjelland Forsaa, Veslemøy Hamre Frantzen, Anita Laugaland, Johannes Lange, Karen Simonsen, Eldbjørg Fiske, Ingvild Dalen, Bernd Müller, Geir Olve Skeie, Marit Renså, Wenche Telstad, Aliaksei Labusau, Jane Kastet, Ineke HogenEsch, Marianne Kjerandsen, Liv Kari Håland, Karen Herlofson, Solgunn Ongre, Siri Bruun, David Burn, Lynn Rochester, Gordon W. Duncan, Tien K. Khoo, Kate S. M. Taylor, Robert Caslake, David J. M. McGhee, Diane Swallow, Joanne Gordon, Clare Harris, Ann Hayman, Nicola Johannesson, Hazel Forbes, Valerie Angus, Alasdair Finlayson, David Dawson, Katie Wilde, David Ritchie, Artur Wozniak, Adrian Martin

**Affiliations:** ^1^ Institute of Applied Health Sciences, University of Aberdeen Aberdeen UK; ^2^ John van Geest Centre for Brain Repair, Department of Clinical Neurosciences University of Cambridge Cambridge UK; ^3^ Newcastle NIHR BRC, Translational and Clinical Research Institute, Newcastle University Newcastle upon Tyne UK; ^4^ Newcastle upon Tyne NHS Foundation Trust Newcastle upon Tyne UK; ^5^ Department of Clinical Science, Neurosciences Umeå University Umeå Sweden; ^6^ The Norwegian Center for Movement Disorders, Stavanger University Hospital Stavanger Norway; ^7^ Department of Clinical Medicine University of Bergen Bergen Norway

## Abstract

**Background:**

An accurate understanding of prognosis in Parkinson's disease (PD) is important for patient information provision, personalized treatment, and clinical trial design, but most previous research has been biased towards younger, healthier patients.

**Objectives:**

To describe key clinical outcomes longitudinally and identify baseline prognostic factors (predictors) for these outcomes using population‐representative PD cohorts.

**Methods:**

We meta‐analyzed individual patient data from six incidence cohorts in Western Europe (Norway, Sweden, and UK). Each cohort aimed to recruit and follow up all newly diagnosed cases in defined population/incidence periods (between 2000 and 2011). We described postural instability (Hoehn & Yahr Stage 3), functional dependency (needing help with daily activities), dementia, and death with up to 12 years' follow‐up and investigated clinical and genetic predictors using frailty Cox models.

**Results:**

In 883 population‐based incident patients, median age at motor symptom onset was 69.2 years. Median time to postural instability and functional dependency was 7.4 years. Dementia affected 49.6% by 10 years and 54.7% had died by 12 years (median survival 9.4 years). Older age, higher Movement Disorder Society‐Unified Parkinson's Disease Rating Scale‐Part III (MDS‐UPDRS‐III) score, and lower Mini‐Mental State Examination (MMSE) were significantly associated with all outcomes; cognitive symptoms and *GBA* polymorphisms with each outcome except mortality; and *APOE* ε4 with increased mortality and dementia.

**Conclusions:**

This first individual patient data meta‐analysis of population‐based incidence cohorts provides robust prognostic data, with fewer selection biases than previous PD studies, for informing people with PD about prognosis. In incidence cohorts, overall PD prognosis is worse than previously suggested, with key outcomes often occurring early. Further work should develop validated prognostic models for objective stratification of prognostic risk and for personalized medicine. © 2026 The Author(s). *Movement Disorders* published by Wiley Periodicals LLC on behalf of International Parkinson and Movement Disorder Society.

Accurate prognostic data in Parkinson's disease (PD) are crucial for informing patients and carers how they may be affected; tailoring treatments to an individual's prognosis; and improving clinical trial design.^1^

Systematic reviews highlighted marked heterogeneity in outcomes across PD cohorts.^2^, ^3^, ^4^ Many factors contribute to this, including varying disease durations at recruitment, different recruitment methods (eg, population‐based studies versus specialist clinic‐based studies), diagnostic inaccuracy, and comorbidity exclusions.^3^ Interpreting prognostic studies is difficult unless participants are recruited at similar times in the disease course, and inception cohorts (studies that recruit and follow‐up patients from diagnosis) are usually the logical way to achieve this objective.^5^ Nevertheless, inception cohort recruitment may be unrepresentative of the general population with PD, for instance, recruiting relatively fewer patients who are older,^6^ frail, or live with comorbidities, factors that strongly influence outcomes. Therefore, the most robust way to study prognosis with the lowest risk of selection bias is long‐term follow‐up of incidence studies (incidence cohorts), which aim to identify and follow all newly diagnosed patients from a defined population and time period. Previous incidence cohorts have been small, hence pooling data from such studies is useful to increase statistical power.

We aimed to perform individual patient data (IPD) meta‐analysis of all known incidence‐based PD cohorts to (1) describe prognosis in terms of key clinical outcomes (postural instability, dependency, dementia, death) and (2) identify baseline prognostic factors (predictors) for these outcomes.

## Methods

### Parkinson's Incidence Cohorts Collaboration (PICC)

In a previous systematic review of incidence studies^6^ we identified PD incidence studies that also had long‐term in‐person follow‐up. The incidence cohorts identified were CamPaIGN, ICICLE‐PD, NYPUM, ParkWest, PICNICS, and PINE.^7^, ^8^, ^9^, ^10^, ^11^, ^12^ Each study began by defining the incidence of PD, seeking to identify all new PD cases in a specified geographical area and recruitment period, using multiple overlapping population‐based case ascertainment methods (all studies regularly asked for referrals from general practitioners, geriatricians, and neurologists, with additional strategies including hand‐searching referral letters, electronic searches of general practice records, and screening of hospital discharge letters). All incidence cases were invited to undergo long‐term follow‐up with regular diagnostic re‐evaluation by neurologists or geriatricians with expertise in movement disorders, guided by the UK PD Brain Bank criteria. We developed a collaborative working agreement forming PICC and common variables were collated in a pooled database. ICICLE‐PD and PICNICS supplemented their incidence cohorts with additional non‐incident inception cases, which were excluded from the current analyses.

### Standard Protocol Approvals, Registrations, and Patient Consents

Each cohort had ethics committee approval and all subjects gave informed consent.

### Prognostic Outcomes

We studied four outcomes:

- Sustained postural instability (Hoehn & Yahr [H&Y] Stage 3)
- Sustained functional dependency, that is, the need for help in basic daily activities using an algorithm derived from the Unified Parkinson's Disease Rating Scale (UPDRS)/Movement Disorder Society (MDS)‐UPDRS (feeding, dressing, hygiene, walking, and arising from chair items).^13^
- Dementia, defined in individual studies either using the Diagnostic and Statistical Manual of Mental Disorders 4th Edition dementia definition or the MDS PD dementia definition.
- All‐cause mortality.

Sustained postural instability/functional dependency was defined as persistence of the outcomes at all subsequent study visits.

### Baseline Prognostic Factors

We reviewed systematic reviews of prognostic factors^2^, ^3^, ^14^ to identify factors with evidence from previous studies. Of those, we had sufficient data in PICC to investigate the following baseline (close to time of diagnosis) prognostic factors:

- Age
- Sex
- Years of education
- Smoking history (current, ex, never)
- Presence of hallucinations (score >1 in UPDRS item 2 or >0 in MDS‐UPDRS item 1.2 except for ICICLE‐PD [any hallucination identified with the North‐East Visual Hallucinations Interview])
- Cognitive symptoms derived from UPDRS item 1/MDS‐UPDRS item 1.1 (0 [none], 1 [cognitive symptoms not impairing functioning], 2+ [cognitive symptoms impairing functioning])
- H&Y disease stage
- Motor impairment: MDS‐UPDRS‐Part III score, converted from UPDRS‐Part III if needed^15^
- Axial impairment: sum of arising from chair, posture, gait, postural instability items from UPDRS/MDS‐UPDRS (recoding of MDS‐UPDRS postural instability item to align with UPDRS)
- Cognitive function: Mini‐Mental State Examination (MMSE) total score (categorized into <27, 27–28, 29, and 30, based on the distribution of MMSE scores in the participants, because its effects were expected to be nonlinear).

We also investigated several genetic variants^16^ as potential prognostic factors based on availability in studies and previous studies of association with prognosis:^8^, ^17^, ^18^

xi Apolipoprotein E (*APOE*) ε4 carrier status
xii Presence of the *MAPT* H1/H1 haplotype
xiii Presence of any glucocerebrosidase (*GBA*) polymorphism. The most common were E326K (6%) and T369M (4%).

### Descriptive Analysis

We calculated median follow‐up using the reverse Kaplan–Meier method.^19^ We described development of the first three outcomes over 10 years and mortality over 12 years. We plotted Kaplan–Meier survival probabilities for each outcome and tabulated these probabilities at 5 and 10 years, median survival, and mean age at outcome. Those who had outcomes already at baseline were excluded from the main survival analyses of the respective outcome. We assessed heterogeneity in the survival distribution between studies using the log‐rank test.

### Prognostic Factor Analysis

We used one‐stage meta‐analysis to investigate baseline prognostic factors for the four previously mentioned outcomes.^20^ We used a shared frailty Cox model which estimates the between‐study frailty term (theta) and adjusts both the point estimates of the hazard ratios and the confidence intervals to account for the additional variability due to heterogeneity between studies.^21^

For each outcome, we entered all the aforementioned prognostic factors into the model. There were more than 10 events per variable in all analyses. Nonlinear relationships between variables and outcomes were investigated using fractional polynomials. The proportional hazards assumption was evaluated using plots of the Schoenfeld residuals and of the log–log of survival function over time. If hazards were non‐proportional, an interaction with time was added to the model and the assumption was retested.

Missing baseline data were assumed missing at random (Little's test, *P* < 0.001, indicated data were not missing completely at random) and were imputed using multiple imputation. Twenty datasets were imputed for each outcome using predictive mean matching and chained equations, with all prognostic factors, the outcome variable, and the Nelson–Aalen estimator of the cumulative hazard function in the imputation models. We also imputed one variable (cognitive symptoms) that was not available from ICICLE‐PD (N = 82). Model estimates were combined using Rubin's rules.

Analyses were performed using Stata 16.

### Systematic Literature Search

We searched Medline to identify previous cohort studies reporting these outcomes in PD, primarily so we could set our results in context in the Discussion section. We identified long‐term studies (at least 3 years of follow‐up) reporting the development of mortality, dementia, functional dependency, or postural instability. We excluded cross‐sectional studies; studies that investigated specific subgroups of PD (eg, those treated with advanced therapies); and those with fewer than 50 participants. We classified previous studies as population‐based, where an attempt was made to identify cases at the population level (ie, in primary as well as secondary/hospital clinic‐based care) and hospital clinic‐based (other cohorts). The search strategy is detailed in Appendix S1.

### Data Sharing

Requests for deidentified patient data can be made to the PICC Steering Committee by contacting the corresponding author.

## Results

Table S1 summarizes the characteristics of the individual cohorts and pooled cohort. Consent to follow‐up among the initial incidence studies was mostly high (78% overall) with 883 cases included in the pooled cohort. Participants' baseline characteristics are shown in Table S2. The pooled median ages at motor onset and diagnosis were 69.2 and 71.0 years, respectively. Age was older than in many previous studies because each cohort sought to include all incident patients. Most participants were White (99%), reflecting the ethnicities of the populations studied. Median follow‐up was 7.3 years, and 5% were lost to follow up for mortality and 14% for other outcomes (see Tables 1 and S2).

**TABLE 1:** Key prognostic outcomes by study in the Parkinson's Incidence Cohorts Collaboration

Parameter	CamPaIGN	ICICLE‐PD	NYPUM	ParkWest	PICNICS	PINE	Pooled cohort
N at baseline	140	82	144	191	125	201	883
N (%) at baseline with^a^							
Postural instability	16 (11)	12 (15)	19 (13)	12 (6)	7 (6)	26 (13)	92 (10)
Dependency	26 (19)	16 (20)	12 (8)	9 (5)	11 (9)	25 (12)	99 (11)
Dementia	0 (0)	0 (0)	0 (0)	1 (1)	0 (0)	4 (2)	5 (1)
N (%) lost to follow‐up							
For vital status	29 (21)	0 (0)	8 (6)	8 (4)	0 (0)	0 (0)	45 (5)
For other outcomes	29 (21)	38 (46)	17 (12)	11 (6)	23 (18)	3 (1.5)	122 (14)
N (%) with outcomes by end of follow‐up^b^							
Postural instability	81 (57)	24 (29)	75 (52)	71 (37)	49 (39)	151 (75)	451 (51)
Functional dependency	95 (67)	34 (41)	80 (56)	73 (38)	48 (39)	138 (69)	468 (53)
Dementia	41 (29)	21 (26)	60 (42)	45 (24)	33 (26)	87 (43)	287 (33)
Death	63 (45)	25 (30)	75 (52)	38 (20)	63 (50)	153 (76)	417 (47)
Kaplan–Meier probability of outcome by 5 years' follow‐up, % (95% CI)							
Postural instability^c^	51.5 (42.3–61.4)	24.4 (14.4–39.4)	24.2 (17.4–33.1)	20.5 (15.1–27.5)	33.7 (25.0–44.4)	45.1 (37.7–53.2)	34.0 (30.6–37.8)^d^
Functional dependency^c^	50.7 (41.1–61.1)	36.2 (24.3–51.6)	27.9 (20.9–36.6)	24.2 (18.4–31.4)	38.7 (28.6–50.9)	44.1 (36.7–52.3)	36.0 (32.4–39.7)^e^
Dementia^c^	20.9 (14.5–29.5)	24.7 (16.3–36.4)	29.7 (22.6–38.5)	20.3 (15.0–27.1)	30.6 (22.3–41.1)	30.1 (23.8–37.7)	26.0 (23.0–29.3)
Death	23.6 (17.3–31.7)	13.5 (7.7–23.1)	20.1 (14.5–27.7)	12.3 (8.4–18.0)	9.6 (5.6–16.3)	28.5 (22.8–35.3)	18.9 (16.5–21.7)
Kaplan–Meier probability of outcome by 10 years’ follow‐up, % (95% CI)							
Postural instability	65.6 (55.8–75.3)	NA	56.0 (45.8–66.7)	NA	NA	84.2 (77.6–89.8)	69.2 (63.8–74.5)^f^
Functional dependency	80.3 (69.7–89.0)	NA	60.8 (51.2–70.5)	NA	NA	79.1 (71.7–85.6))	70.7 (65.5–75.7)^g^
Dementia	39.8 (30.7–50.5)	NA	54.0 (43.6–64.0)	NA	NA	57.5 (49.4–65.8)	49.6 (44.6–54.8)
Death	49.3 (40.4–58.9)	NA	55.8 (47.0–65.1)	NA	42.4 (34.3–61.5)	66.3 (59.7–72.8)	54.7 (50.6–68.9)
Median time in years (95% CI) to							
Postural instability	4.8 (2.8–6.3)	NR	9.5 (7.5–ND)	NR	7.2 (5.5–ND)	5.5 (4.5–5.6)	7.4 (6.5–7.6)^h^
Functional dependency	4.5 (3.8–5.9)	NR (3.8–ND)	7.6 (7.4–9.6)	NR	7.5 (4.7–ND)	5.6 (4.5–7.5)	7.4 (6.4–7.5)^i^
Dementia	NR (8.0–ND)	NR (7.5–ND)	8.5 (7.5–ND)	NR	NR (8.8–ND)	8.3 (6.6–ND)	NR (8.5–ND)
Death	10.1 (7.9–ND)	8.1 (6.8–ND)	9.8 (8.6–10.5)	NR	11.5 (9.8–ND)	7.7 (6.9–8.5)	9.4 (9.0–ND)
Mean age in years (SD) at							
Motor symptom onset	68.3 (10.0)	64.9 (10.8)	69.4 (9.8)	65.8 (9.3)	67.4 (9.8)	70.2 (10.4)	67.8 (10.1)
Median age at diagnosis	70.3 (9.6)	66.6 (10.6)	71.2 (9.9)	67.9 (9.3)	68.7 (9.6)	72.1 (10.4)	69.8 (10.0)
Postural instability	75.4 (7.8)	77.8 (5.6)	77.8 (7.2)	76.9 (6.5)	74.1 (8.3)	77.8 (7.4)	76.8 (7.4)
Functional dependency	74.2 (8.3)	74.1 (8.2)	77.7 (6.9)	75.7 (6.5)	73.8 (7.6)	79.0 (7.0)	76.5 (7.5)
Dementia	78.9 (6.7)	78.6 (6.8)	77.7 (7.1)	77.3 (6.0)	74.0 (7.9)	80.1 (7.0)	78.2 (7.1)
Death	79.5 (7.7)	78.9 (8.8)	82.8 (6.2)	78.9 (6.5)	81.7 (7.4)	81.8 (8.3)	81.3 (7.7)

^a^ Excluded from main survival analyses.

^b^ Maximum 10 years for each outcome except death, where maximum was 12 years.

^c^ At 4.5 years in ICICLE‐PD.

^d^ 40.9 (37.5–44.5) if those with outcome at baseline are included.

^e^ 43.2 (39.7–46.8) if those with outcome at baseline are included.

^f^ 72.5 (67.5–77.2) if those with outcome at baseline are included.

^g^ 74.0 (69.3–78.4) if those with outcome at baseline are included.

^h^ 6.5 (5.6–6.8) if those with outcome at baseline are included.

^i^ 6.0 (5.5–6.5) if those with outcome at baseline are included.

Abbreviations: CI, confidence interval; NA, not available; ND, not defined (insufficient follow‐up time to define the upper bound of confidence interval); NR, median time not reached (fewer than 50% developed the outcome by the end of follow‐up); SD, standard deviation.

### Heterogeneity

**FIG. 1:**
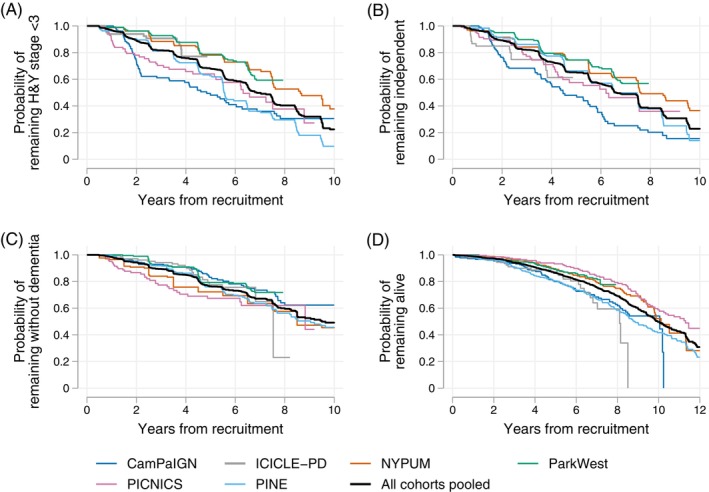
Kaplan–Meier probabilities of outcomes over time in the Parkinson's Incidence Cohorts Collaboration, in all cohorts pooled and by each cohort. (A) Probability of remaining Hoehn and Yahr (H&Y) stage <3. (B) Probability of remaining functionally independent. (C) Probability of remaining free from dementia. (D) Probability of remaining alive. [Color figure can be viewed at wileyonlinelibrary.com]

For each outcome there was substantial between‐cohort heterogeneity, though less for dementia and death (Fig. 1, log‐rank test *P* < 0.001 for postural instability, dependency, and death; *P* = 0.02 for dementia). There was little heterogeneity between cohorts for predictors (the frailty terms were all small [≤0.14] and not significantly different from zero).

### Postural Instability

Ninety‐two participants (10%) had sustained postural instability from baseline. Some 359 patients developed sustained postural instability after baseline with median time to postural instability of 7.4 years (95% confidence interval [95% CI] 6.5–7.6) and 10‐year probability of postural instability was 69.2% (95% CI 63.8–74.5) (Table 1).

Significant baseline predictors of postural instability were age (hazard ratio [HR] for 10‐year increase = 2.61 [95% CI 2.23–3.05]); female sex (time‐varying effect, HR up to 6 years = 1.05 [95% CI 0.81–1.36], HR 6–10 years = 2.82 [95% CI 1.81–4.39]); years of education (HR = 1.05 [95% CI 1.01–1.08]); presence of cognitive symptoms (HR for cognitive symptoms impairing functioning vs. none = 2.59 [95% CI 1.52–4.40]); MDS‐UPDRS‐III (HR for 10‐point increase = 1.36 [95% CI 1.22–1.51]); MDS‐UPDRS axial score (HR 1.30 [95% CI 1.24–1.38]); lower MMSE (HR 29 vs. 30 = 1.59 [95% CI 1.17–2.15], HR 27/28 vs. 30 = 1.42 [95% CI 1.04–1.92]); and any *GBA* mutation (HR = 1.72 [95% CI 1.23–2.41]) (Table 2).

**TABLE 2:** Prognostic factors for key outcomes in the Parkinson's Incidence Cohorts Collaboration

Baseline prognostic factor	Outcome							
	Time to sustained postural instability (H&Y Stage 3) (N = 741)^a^		Time to sustained dependency (N = 742)^b^		Time to dementia (N = 853)^c^		Time to death (N = 880)^d^	
	HR (95% CI)	*P*‐value	HR (95% CI)	*P*‐value	HR (95% CI)	*P*‐value	HR (95% CI)	*P*‐value
Age at baseline (HR for 10‐year increase)	**2.61 (2.23–3.05)**	<0.001	**2.05 (1.77–2.37)**	<0.001	**1.93 (1.63–2.28)**	<0.001	**2.44 (2.11–2.82)**	<0.001
Female vs. male sex			0.94 (0.75–1.18)	0.60	1.19 (0.92–1.54)	0.18	**0.74 (0.59–0.91)**	0.005
Female vs. male Up to 6 years; Year 6 onwards	1.05 (0.81–1.36) **2.82 (1.81–4.39)**	0.72 <0.001						
Years of education	**1.05 (1.01–1.08)**	0.005	1.03 (1.00–1.06)	0.08	1.02 (0.98–1.06)	0.31	1.03 (0.99–1.06)	0.11
Current vs. never smoker	1.34 (0.83–2.17)	0.24	1.19 (0.73–1.94)	0.49	0.97 (0.55–1.71)	0.92	1.38 (0.86–2.22)	0.18
Ex vs. never smoker	0.99 (0.78–1.26)	0.95	1.01 (0.80–1.28)	0.91	0.87 (0.66–1.14)	0.30	1.21 (0.98–1.50)	0.08
Presence of hallucinations	1.44 (0.80–2.60)	0.22	**2.14 (1.13–4.07)**	<0.02	**2.58 (1.45–4.29)**	0.001	**1.54 (1.01–2.34)**	0.04
Cognitive symptoms not impairing functioning vs. no cognitive symptoms	1.23 (0.96–1.58)	0.10	**1.38 (1.08–1.75)**	0.01	**1.73 (1.30–2.31)**	<0.001	1.06 (0.84–1.34)	0.65
Cognitive symptoms impairing functioning vs. no cognitive symptoms	**2.59 (1.52–4.40)**	<0.001	1.78 (0.98–3.21)	0.06	**2.44 (1.39–4.29)**	0.002	1.39 (0.91–2.12)	0.13
H&Y stage	1.14 (0.92–1.41)	0.24	1.01 (0.82–1.26)	0.90	1.12 (0.91–1.38)	0.30	**1.21 (1.02–1.43)**	0.03
MDS‐UPDRS‐III (HR for 10‐point increase)	**1.36 (1.22–1.51)**	<0.001	**1.30 (1.18–1.45)**	<0.001	**1.27 (1.13–1.43)**	<0.001	**1.22 (1.11–1.34)**	<0.001
Axial severity score^e^	**1.30 (1.24–1.38)**	<0.001	**1.18 (1.12–1.25)**	<0.001	**1.11 (1.04–1.18)**	0.003	**1.05 (1.00–1.10)**	0.045
MMSE: 29 vs. 30	**1.59 (1.17–2.15)**	0.003	**1.36 (1.01–1.85)**	0.05	1.34 (0.91–1.97)	0.14	1.08 (0.81–1.47)	0.58
MMSE: 27/28 vs. 30	**1.42 (1.04–1.92)**	0.03	**1.63 (1.20–2.21)**	0.002	**2.09 (1.44–3.02)**	<0.001	**1.43 (1.07–1.90)**	0.02
MMSE: <27 vs. 30	1.36 (0.90–2.05)	0.14	**2.17 (1.44–3.26)**	<0.001	**3.32 (2.12–5.20)**	<0.001	**1.55 (1.09–2.22)**	0.02
APOE ε4 carrier	1.14 (0.89–1.46)	0.31	0.98 (0.75–1.28)	0.86	**2.14 (1.59–2.89)**	<0.001	**1.42 (1.12–1.80)**	0.004
MAPT H1/H1 haplotype	1.05 (0.82–1.35)	0.70	0.86 (0.68–1.10)	0.23	1.10 (0.83–1.44)	0.51	1.08 (0.86–1.36)	0.50
Any GBA mutation	**1.72 (1.23–2.41)**	0.002	**1.79 (1.26–2.52)**	0.001	**2.18 (1.50–3.16)**	<0.001	1.02 (0.70–1.50)	0.91

*Note*: Multivariable models adjusted for all other listed variables (except for axial severity score which was entered into a model without MDS‐UPDRS‐II score). Univariable analyses are reported in Table S3. Hazard ratios in bold type represent significant prognostic factors.

^a^ Ninety‐two participants had sustained postural instability from baseline and were excluded and 50 participants had no follow‐up data for this outcome (14 due to death before the first follow‐up visit and 36 due to loss to follow‐up).

^b^ Three participants had no data available for this variable, 99 participants had sustained dependency from baseline, and 39 participants had no follow‐up data for this outcome (12 due to death before first follow‐up visit and 27 due to loss to follow‐up).

^c^ Five participants had dementia at baseline and were excluded; 25 participants had missing dementia data due to loss to follow‐up for this outcome.

^d^ Three participants had missing data for death due to loss to follow‐up.

^e^ Sum of axial items in MDS‐UPDRS‐III or original UPDRS (sum of arising from chair, posture, gait, and postural instability items).

Abbreviations: H&Y, Hoehn & Yahr; HR, hazard ratio; CI, confidence interval; MDS‐UPDRS, Movement Disorder Society‐Unified Parkinson's Disease Rating Scale; MMSE, Mini‐Mental State Examination; APOE, apolipoprotein E; MAPT, microtubule‐associated protein tau; GBA, glucocerebrosidase.

### Dependency

Ninety‐nine participants (11%) were dependent at baseline. Some 369 participants developed sustained functional dependency during follow‐up. Median time to functional dependency was 7.4 years (95% CI 6.4–7.5) and the 10‐year probability was 70.7% (95% CI 65.5–75.7) (Table 1).

Independent predictors of dependency were age (HR for 10‐year increase = 2.05 [95% CI 1.77–2.37]); presence of hallucinations (HR = 2.14 [95% CI 1.13–4.07]); presence of cognitive symptoms (HR for cognitive symptoms not impairing functioning vs. none = 1.38 [95% CI 1.08–1.75]); MDS‐UPDRS‐III (HR for 10‐point increase = 1.30 [95% CI 1.18–1.45]); MDS‐UPDRS axial score (HR 1.18 [95% CI 1.12–1.25]); lower MMSE (HR 29 vs. 30 = 1.36 [95% CI 1.01–1.85]), HR 27/28 vs. 30 = 1.63 [95% CI 1.20–2.21]), HR <27 vs. 30 = 2.17 [95% CI 1.44–3.26]); and any *GBA* mutation (HR = 1.79 [95% CI 1.26–2.52]) (Table 2).

### Dementia

Five participants (1%) had dementia at baseline (delayed PD diagnoses). Some 282 participants (32%) developed dementia during follow‐up. The 10‐year dementia probability was 49.6% (95% CI 44.6–54.8) (Table 1).

Independent predictors of dementia were age (HR for 10‐year increase = 1.93 [95% CI 1.63–2.28]); presence of hallucinations (HR = 2.58 [95% CI 1.45–4.29]); presence of cognitive symptoms (HR for cognitive symptoms not impairing functioning vs. none = 1.73 [95% CI 1.30–2.31], HR for cognitive symptoms impairing functioning vs. none = 2.44 [95% CI 1.39–4.29]); MDS‐UPDRS‐III (HR for 10‐point increase = 1.27 [95% CI 1.13–1.43]); MDS‐UPDRS axial score (HR 1.11 [1.04–1.18]); lower MMSE (HR 27/28 vs. 30 = 2.09 [95% CI 1.44–3.02], HR <27 vs. 30 = 3.32 [95% CI 2.12–5.20]); *APOE* ε4 status (HR = 2.14 [95% CI 1.59–2.89]); and any *GBA* mutation (HR = 2.18 [95% CI 1.50–3.16]) (Table 2).

### Death

A total of 415 patients (47%) died during follow‐up up to 12 years. Median time to death was 9.4 years. The 10‐year mortality probability was 54.7 (95% CI 50.6–68.9) (Table 1).

The independent predictors of death were age (HR for 10‐year increase = 2.44 [95% CI 2.11–2.82]); male sex (HR = 1.35 [95% CI 1.10–1.69]); hallucinations (HR = 1.54 [95% CI 1.01–2.34]); H&Y stage (HR = 1.21 [95% CI 1.02–1.43]); MDS‐UPDRS‐III score (HR for 10‐unit increase = 1.22 [95% CI 1.11–1.34]); MDS‐UPDRS axial score (HR 1.22 [1.11–1.34]); lower MMSE (HR for MMSE 27/28 vs. 30 = 1.43 [95% CI 1.07–1.90], HR for MMSE <27 vs. 30 = 1.55 [95% CI 1.09–2.22]); and *APOE* ε4 status (HR = 1.42 [95% CI 1.12–1.80]) (Table 2).

Proportional hazards assumptions were satisfied (with a sex–time interaction in the postural instability model). Table S3 reports univariable associations with outcomes.

### Age‐Stratified Probabilities

Given the importance of baseline age in defining subsequent risk of poor outcome, age‐stratified Kaplan–Meier probabilities of outcomes are presented in Tables 3 and S4.

**TABLE 3:** Probabilities of developing four key outcomes in Parkinson's disease at 5 and 10 years from diagnosis, by age stratum

Age stratum (years)	Postural instability^a^ (men and women)	Functional dependency^b^ (men and women)	Dementia (men and women)	Mortality in men	Mortality in women
	Percentage with outcome at 5 years from diagnosis				
<50	0	0	0	3^c^	3^c^
50–59	9	12	6	3^c^	3^c^
60–69	20	21	16	8	8
70–79	47	50	35	23	18
80+	72	70	50	54	41
	Percentage with outcome at 10 years from diagnosis				
<50	10	28	6	8	7
50–59	34	39	20	22	9
60–69	56	63	46	38	24
70–79	85	87	62	75	63
80+	100	100	74	91	84

^a^ In those without postural instability at baseline.

^b^ In those independent at baseline.

^c^ Proportions of deaths averaged in men and women due to small numbers of deaths in age strata <60 years at 5 years. Actual values were 0% in men <50 years, 7% in women <50 years, 5% in men 50–59 years, and 0% in women 50–59 years.

### Systematic Literature Search

**FIG. 2:**
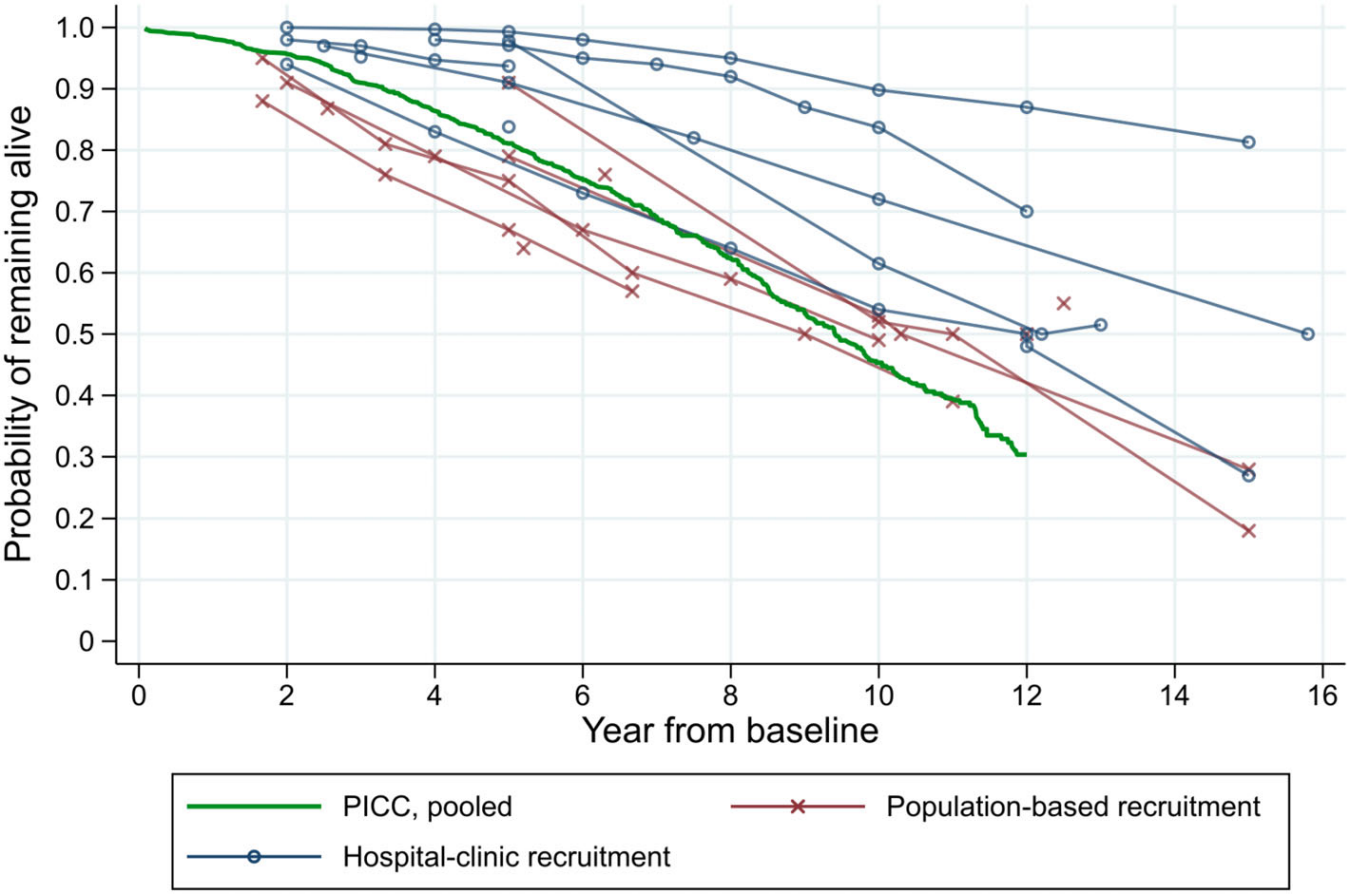
Comparison of survival probabilities over time in previous inception studies and the current study. The orange line plots the Kaplan–Meier survival probabilities from the pooled Parkinson's Incidence Cohorts Collaboration (PICC) cohort. Crosses mark survival estimates from previous inception studies with population‐based recruitment and open circles mark survival estimates from previous inception studies with hospital clinic‐based recruitment. Values from previous studies are either those reported explicitly in the published paper or are estimated from Kaplan–Meier plots. Connecting lines link multiple survival points from individual studies where available and single survival points represent studies where survival was only reported at a single timepoint. [Color figure can be viewed at wileyonlinelibrary.com]

Tables S5 and S6 contain a summary of studies identified in the Medline search. Figure 2 shows a comparison with previous inception studies of survival in PD by recruitment method. Age at diagnosis was higher in population‐based studies (weighted mean 68.7 years [95% CI 66.3–71.0]) than in hospital‐based studies (weighted mean 63.1 years [95% CI 61.3–64.9]). Average estimates of survival in hospital clinic‐based studies were substantially higher than those in population‐based studies. At 5 and 10 years, respectively, inverse‐variance weighted mean survival in hospital‐based studies was 98% (95% CI 95–100) and 72% (95% CI 53–91) and in population‐based studies was 73% (95% CI 56–90) and 50% (95% CI 44–55).

## Discussion

We present the first IPD meta‐analysis of key PD outcomes and their baseline predictors using population‐based incidence cohorts. We demonstrate that poor outcomes in PD are common and often occur early (eg, about 10% had postural instability and dependency at diagnosis and over 25% developed dementia within 5 years). These unselected population data from incident patients, especially age‐stratified findings, can guide discussions regarding prognosis in PD in predominantly Caucasian high‐income countries. We confirmed that age, motor severity, cognitive impairment, *APOE* ε4, and *GBA* variants are important prognostic factors and identified a novel association between *APOE* and higher mortality. After confounder adjustment, H&Y stage was only weakly associated with mortality and smoking showed no significant associations.

### Comparison with Previous Studies (Prognostic Outcomes)

Previous publications from individual PICC cohorts reporting these outcomes are summarised in Table S7. A Californian health maintenance organization, comprising 25–30% of the general population, with similar demographics to the general population, defined PD incidence^22^ and studied mortality. In 573 cases, the median survival was 9 years,^23^ similar to our findings, but other long‐term outcomes were not reported.

There is striking heterogeneity in previous mortality studies in PD, but inception studies have been more consistent than non‐inception studies.^3^ We compared our results with 24 previous inception studies reporting survival. Median survival times in population‐based inception studies ranged from 9 to 12 years, similar to our findings.^24^, ^25^, ^26^ Additionally, other survival probabilities in such studies were similar to our study (24% mortality at 6.3 years^27^ and 55% mortality at 12.5 years).^28^ Population‐based studies in Israel reported higher mortality,^29^, ^30^ possibly relating to diagnostic inaccuracies based on drug claims or genetic differences. Hospital (specialist) clinic‐based inception cohorts not only have greater heterogeneity and tend to have lower mortality (Fig. 2). Several had median survival around 12 years,^31^, ^32^, ^33^ but one study reported a median of 15.8 years^34^ and others 10‐year mortalities of 10% and 16%.^35^, ^36^

We hypothesize that the substantial differences in mortality risk (and other outcomes) between population‐based and hospital‐based studies is because hospital‐based studies are likely to under‐recruit people with PD who are elderly or frail and therefore at higher risk of poor outcomes, and are less likely to be referred to a movement disorders specialist in many healthcare settings. Many seminal studies in PD recruited from specialist movement disorders clinics and so are unlikely to be generalizable to wider PD populations. The median time for motor symptom onset to recruitment to our study was 1.4 years and so delayed diagnosis is not the reason for shorter survival times.

While dementia in PD has been widely studied, few population‐based inception cohorts exist. Non‐population‐based inception cohorts consistently reported lower dementia incidence than ours. Some, only slightly lower than our findings, were presumably more representative of their population. For example, a nationwide Taiwanese insurance claims study and the Sydney Multicentre Study reported 10‐year Kaplan–Meier probabilities of dementia of 43% and 45%, respectively.^33^, ^37^ Other inception studies reported substantially lower dementia incidences. A United States (US) population‐based inception study reported the risk of MMSE <24 as 15% after 5.7 years’ follow‐up.^28^ Two UK studies of early PD reported 7% and 12% of patients developing dementia at 3.7 and 4.5 years.^38^ While these three studies did not account for censoring (underestimating dementia risk), relative under‐recruitment of older frailer patients may lead to both lower average onset age and lower dementia risk. The PPMI study reported that the 10‐year probability of dementia was only 9%,^39^ which is dramatically lower than in population‐representative studies such as ours. Some non‐inception studies with less rigorous recruitment methods have reported much lower risk of dementia than any of the inception studies (Table S5).

This is the largest study of functional dependency in PD to date. Previous inception studies reported lower dependency risk than we found,^2^, ^33^, ^36^, ^40^ but none were based on incidence studies and so these differences may again be due to selection bias. Non‐inception hospital‐based studies have generally found lower risk of dependency than we report, despite longer average disease durations at recruitment.^41^, ^42^

Few studies have reported longitudinal development of postural instability. One Chinese hospital‐based inception study reported a median time to H&Y Stage 3 of 8.3 years^43^ and a US non‐inception study reported 37% of participants having falls within 5 years from disease onset^41^ – both lower than our findings.

### Comparison with Previous Studies (Prognostic Factors)

We confirm previous work associating worse prognosis with older age, greater parkinsonian impairment, and poorer cognition at diagnosis; higher risk of dementia in *APOE* ε4 allele carriers; and poorer prognosis in *GBA* mutation carriers,^2^, ^14^, ^16^ including previous work from our cohorts (Table S7). Together with previous inception studies, this study provides strong evidence that mortality in men is increased compared with women (Table S8), possibly reflecting higher mortality in men in general.

*APOE* ε4 carriers showed a small, but statistically significant, increased mortality risk, which has not, to our knowledge, previously been reported in PD, though it has been reported in other populations. This may be mediated by increased dementia risk in *APOE* ε4 carriers.^17^ Another notable finding is that subjective cognitive symptoms independently predicted dementia after adjustment for MMSE, adding to recent interest in subjective cognitive decline in PD and Alzheimer's disease,^44^ although further investigation with better cognitive instruments would be useful. We could not confirm *MAPT* H1/H1 haplotype as a predictor of prognosis, although previously reported by one cohort included in this meta‐analysis.^8^

Early disease predictors of functional dependency and postural instability have rarely been studied previously.^45^, ^46^, ^47^ Other than individual studies included in this IPD meta‐analysis, only one inception study previously studied predictors of dependency, and only reported on obesity.^46^

### Heterogeneity

Although there was significant heterogeneity in the descriptive analyses, there was substantially lower heterogeneity between the incidence cohorts for both the dementia and death outcomes than for the other outcomes, even though previous systematic reviews have highlighted between‐study heterogeneity in these outcomes.^3^, ^4^ Possibly much of the heterogeneity in previous studies was due to varying recruitment methods resulting in heterogeneous distribution of prognostic factors, such as age and comorbidity, between studies. Some of the heterogeneity in our studies may reflect real between‐population differences but may also reflect differences in the assessment of H&Y status or UPDRS activities of daily living questions, given the subjective elements in the scales. It may also relate to different follow‐up frequencies, as longer intervals between follow‐ups may introduce bias due to later determination of outcomes, on average. Furthermore, the heterogeneity between cohorts in the analysis of predictors was low for all outcomes, with non‐significant frailty terms, and so much of the heterogeneity between studies is likely to be due to the variables we included in the survival modeling. Future work with meta‐regression in a larger sample of cohorts could investigate this further.

### Strengths

We included all the incidence cohorts worldwide that we identified in a previous systematic review, which should minimize bias in the selection of studies for this IPD meta‐analysis. The combination of the large sample size and the use of population‐based incidence cohorts arising from rigorous methods to identify and follow up as many new patients with PD as possible makes this a unique cohort for studying the epidemiology and prognosis of PD. These methods, together with the low attrition rate, mean that the selection bias will be lower than in most other studies, which may under‐recruit those individuals who are older, frailer, have more comorbidities, or have lower socioeconomic status.^48^

Studying these patients from the time of diagnosis is the optimal way to investigate prognosis and avoids selection biases inherent in non‐inception cohorts, such as omission of those who die early in the disease course, and over‐representation of long‐duration PD. Each included study had in‐person follow‐up with comprehensive data collection, used home visits where clinic attendance was difficult, and had a relatively long follow‐up period. We used rigorous IPD meta‐analysis methods, multiple imputation, and adjustment for between‐study heterogeneity.

### Limitations

Common data on certain potential prognostic factors were not available across the cohorts (eg, specific cognitive features, rapid eye movement [REM] sleep behavior disorder, comorbidity, frailty). We did not adjust for multiple comparisons, because many of the analyses were not independent of each other. We lacked statistical power to investigate rare genetic variables such as individual *GBA* polymorphisms, LRRK2, or PRKN mutations. We did not investigate treatment‐related variables as most participants were untreated at baseline. There were few young‐onset patients due to the low incidence of young‐onset PD. Most participants were White, and all were in high‐income countries, so replication in other populations is needed. Fewer than 50% of participants died by the end of the available follow‐up, so median time to death may change slightly with further follow‐up. Our studies predate the MDS PD diagnostic criteria, but we have used expert clinical diagnosis guided by the UK Brain Bank Criteria, with repeated reassessment over follow‐up to reduce misdiagnosis. Finally, MMSE has limitations in PD, including ceiling effects, but this was the only common cognitive measure in our cohorts.

### Further Work

Updating our data after longer follow‐up will be important. Incidence cohorts in non‐White populations are needed. While we provide useful data to inform discussions with patients, further development of prognostic models (tools that combine prognostic factors to predict future outcomes) will enable tailored individualized estimates of prognosis.^49^, ^50^ Prognostic models also have important benefits for research, including incorporation into clinical trial design to improve trial efficiency.^1^ This work has focused on baseline predictors but investigating change in these factors over time or factors more prevalent later in the disease course would be useful. Discussing prognosis with patients can be difficult, so research should explore how best to do this sensitively while providing hope.

## Conclusions

This IPD meta‐analysis of population‐based incidence cohorts provides the best available data for informing people with sporadic PD about prognosis, with fewer selection biases than most previous studies, by explicitly seeking to recruit all cases of PD in the population. These data strongly suggest that prognosis in PD is worse than has often been suggested in less representative studies. Our comparison with previous studies of prognosis in PD suggests that study design influences the reported outcomes. We therefore recommend that future studies of prognosis of PD in general study patients from early in the disease course and try to recruit population‐representative samples. Caution is needed when generalizing hospital‐based studies of prognosis. Further work is needed to develop validated prognostic models for objective risk stratification for prognostication and personalized medicine.

## Author Roles

(1) Research Project: A. Design, B. Organization, C. Execution; (2) Statistical Analysis: A. Design, B. Execution, C. Review and Critique; (3) Manuscript Preparation: A. Writing of the First Draft, B. Editing of the Final Version.

A.D.M: 1A, 1B, 1C, 2A, 2B, 3A.

D.J.M.: 2B, 3B.

M.C.: 1A, 1C, 3B.

C.H.W.‐G.: 1A, 1C, 3B.

R.A.L.: 1A, 1C, 3B.

A.J.Y: 1A, 1C, 3B.

D.B.: 1A, 1C, 3B.

L.F.: 1A, 1C, 3B.

J.M.‐G.: 1A, 1C, 3B.

G.A.: 1A, 1C, 3B.

O.‐B.T.: 1A, 1C, 3B.

C.E.C.: 1A, 1C, 3B.

## Disclosures

A.D.M. reports research funding from the Chief Scientist Office of the Scottish Government, Parkinson's UK, the Academy of Medical Sciences, LifeArc, National Institute of Health and Social Care Research, and NHS Grampian Charity. D.J.M. reports research funding from the Chief Scientist Office of the Scottish Government, NHS Grampian Endowments, Tenovus Scotland and Medical Research Scotland; honoraria from Merck, European Society of Human Reproduction, and Embryology and Reproductive BioMed Online; travel expenses from Merck, Royal Statistical Society, and European Society of Human Reproduction and Embryology. M.C, reports support by the Cambridge Centre for Parkinson's Plus and funding from the Evelyn Trust and Parkinson's UK. C.H.W.‐G. reports research funding from the Medical Research Council (MRC), NIHR Cambridge Biomedical Research Centre (NIHR203312), Cure Parkinson's, Parkinson's UK, Cambridge Centre for Parkinson‐Plus; honoraria from Profile Pharma Limited and GSK; advisor fees from The Michael J. Fox Foundation; consultancy fees from Modus Outcomes, Evidera, Helicon Bio Ltd, and Mission Therapeutics; travel stipends from the World Parkinson Congress, Movement Disorder Society, International Society for Neurochemistry, and the European Cooperation in Science and Technology. R.A.L. reports support by a Janet Owens Parkinson's UK Senior Research Fellowship (F‐1801); grants from Lewy Body Society; and travel expenses from The Michael J. Fox Foundation. A.J.Y. reports grant funding from Dunhill Medical Trust, European Union (EU) IMI, NIHR, Parkinson's UK, The Michael J. Fox Foundation, Weston Brain Institute, Intercept pharmaceuticals, Lewy Body Society, Cure Parkinson's Trust; support and/or honoraria from Britannia, UCB, AbbVie, GSK, and Teva‐Lundbeck for attending conferences. D.B. reports no disclosures relevant to the article. L.F. reports research funding from the Swedish Medical Research Council, Erling Persson Foundation, Parkinson Foundation in Sweden, Umeå University, King Gustaf V and Queen Victoria Freemason Foundation, and the Swedish Parkinson Foundation. J.M.‐G. reports research funding from the Norwegian Parkinson Research Foundation. G.A. reports research funding from the Norwegian Parkinson Research Foundation, Rebergs Legacy, Western Norway Regional Health Trust, and Research Council of Norway. O.‐B.T. reports no disclosures relevant to the manuscript. C.E.C. reports research funding from Parkinson's UK, NHS Grampian Endowments, and RS Macdonald Trust.

## Supporting information


**Appendix S1.** Medline systematic literature search strategy.
**Table S1.** Characteristics of incidence cohorts included in the Parkinson's Incidence Cohorts Collaboration.
**Table S2.** Participants' baseline characteristics.
**Table S3.** Prognostic factors for key outcomes in the Parkinson's Incidence Cohorts Collaboration (univariable and multivariable analysis).
**Table S4.** Probabilities of developing four key outcomes in Parkinson's disease at 5 and 10 years from diagnosis, by age stratum.
**Table S5.** Results of systematic search of published literature – previous studies reporting longitudinal outcomes (postural instability, functional dependency, dementia, and mortality).
**Table S6.** Results of systematic search of published literature – previous studies reporting prognostic factors for functional dependency, dementia, and mortality.
**Table S7.** Previous Parkinson's Incidence Cohorts Collaboration publications on dependency, postural instability dementia, and mortality.
**Table S8.** Association between sex and mortality in inception studies in Parkinson's disease and Supplemental References.

## Data Availability

The data that support the findings of this study are available on request from the corresponding author. The data are not publicly available due to privacy or ethical restrictions.

## Appendix A List of Co‐Investigators

Thomas Foltynie, University College London, UK, Co‐Investigator, CamPaIGN cohort, setting up cohort and data collection; Sarah L. Mason, University of Cambridge, UK, Co‐Investigator, CamPaIGN and PICNICS cohorts, data collection; Ruwani Wijeyekoon, University of Cambridge, UK, Co‐Investigator, PICNICS cohort, data collection; Jonathan Evans, Nottingham University Hospital, UK, Co‐Investigator, PICNICS cohort, setting up cohort and data collection; David P. Breen, University of Edinburgh, UK, Co‐Investigator, PICNICS cohort, data collection; Gemma Cummins, University of Cambridge, UK, Co‐Investigator, PICNICS cohort, data collection; Krista Farrell, University of Cambridge, UK, Co‐Investigator, PICNICS cohort, data collection; Kirsten Scott, University of Cambridge, UK, Co‐Investigator, PICNICS cohort, data collection; Tom Stoker, University of Cambridge, UK, Co‐Investigator, PICNICS cohort, data collection; Julia Greenland, University of Cambridge, UK, Co‐Investigator, PICNICS cohort, data collection; Natalie Valle Guzman, University of Cambridge, UK, Co‐Investigator, PICNICS cohort, data collection; Lucy Collins, University of Cambridge, UK, Co‐Investigator, PICNICS cohort, data collection; Simon Stott, University of Cambridge, UK, Co‐Investigator, PICNICS cohort, data collection; Jan Linder, Umeå University, Sweden, Co‐Investigator, NYPUM cohort, study design, data collection; Mona Edström, Umeå University, Sweden, Co‐Investigator, NYPUM cohort, data collection; Jörgen Andersson, Umeå University, Sweden, Co‐Investigator, NYPUM cohort, data collection; Linda Eriksson, Umeå University, Sweden, Co‐Investigator, NYPUM cohort, data collection; Gun‐Marie Hariz, Umeå University, Sweden, Co‐Investigator, NYPUM cohort, data collection; Magdalena Domellöf, Umeå University, Sweden, Co‐Investigator, NYPUM cohort, data collection; Michaela Dreetz Gjerstad, Stavanger University Hospital, Norway, Co‐Investigator, ParkWest cohort, data collection; Kenn Freddy Pedersen, Stavanger University Hospital, Norway, Co‐Investigator, ParkWest cohort, data collection; Elin Bjelland Forsaa, Stavanger University Hospital, Norway, Co‐Investigator, ParkWest cohort, data collection; Veslemøy Hamre Frantzen, Stavanger University Hospital, Norway, Co‐Investigator, ParkWest cohort, data collection; Anita Laugaland, Stavanger University Hospital, Norway, Co‐Investigator, ParkWest cohort, data collection; Johannes Lange, Stavanger University Hospital, Norway, Co‐Investigator, ParkWest cohort, data collection; Karen Simonsen, Stavanger University Hospital, Norway, Co‐Investigator, ParkWest cohort, data collection; Eldbjørg Fiske, Stavanger University Hospital, Norway, Co‐Investigator, ParkWest cohort, data collection; Ingvild Dalen, Stavanger University Hospital, Norway, Co‐Investigator, ParkWest cohort, data collection; Bernd Müller, Haukeland University Hospital, Norway, Co‐Investigator, ParkWest cohort, data collection; Geir Olve Skeie, Haukeland University Hospital, Norway, Co‐Investigator, ParkWest cohort, data collection; Marit Renså, Haukeland University Hospital, Norway, Co‐Investigator, ParkWest cohort, data collection; Wenche Telstad, Førde Hospital, Norway, Co‐Investigator, ParkWest cohort, data collection; Aliaksei Labusau, Førde Hospital, Norway, Co‐Investigator, ParkWest cohort, data collection; Jane Kastet, Førde Hospital, Norway, Co‐Investigator, ParkWest cohort, data collection; Ineke HogenEsch, Haugesund Hospital, Norway, Co‐Investigator, ParkWest cohort, data collection; Marianne Kjerandsen, Haugesund Hospital, Norway, Co‐Investigator, ParkWest cohort, data collection; Liv Kari Håland, Haugesund Hospital, Norway, Co‐Investigator, ParkWest cohort, data collection; Karen Herlofson, Sørlandet Hospital Arendal, Norway, Co‐Investigator, ParkWest cohort, data collection; Solgunn Ongre, Sørlandet Hospital Arendal, Norway, Co‐Investigator, ParkWest cohort, data collection; Siri Bruun, Sørlandet Hospital Arendal, Norway, Co‐Investigator, ParkWest cohort, data collection; David Burn, University of Newcastle, UK, Co‐Investigator, ICICLE‐PD cohort, Chief Investigator, setting up of study; Lynn Rochester, University of Newcastle, UK, Co‐Investigator, ICICLE‐PD cohort, setting up of study; Gordon W. Duncan, University of Newcastle, UK, Co‐Investigator, ICICLE‐PD cohort, data collection; Tien K. Khoo, University of Newcastle, UK, Co‐Investigator, ICICLE‐PD cohort, setting up of study, data collection; Kate S. M. Taylor, University of Aberdeen, UK, Co‐Investigator, PINE cohort, data collection; Robert Caslake, University of Aberdeen, UK, Co‐Investigator, PINE cohort, data collection; David J. M. McGhee, University of Aberdeen, UK, Co‐Investigator, PINE cohort, data collection; Diane Swallow, University of Aberdeen, UK, Co‐Investigator, PINE cohort, data collection; Joanne Gordon, University of Aberdeen, UK, Co‐Investigator, PINE cohort, data collection; Clare Harris, University of Aberdeen, UK, Co‐Investigator, PINE cohort, data collection; Ann Hayman, University of Aberdeen, UK, Co‐Investigator, PINE cohort, data collection; Nicola Johannesson, University of Aberdeen, UK, Co‐Investigator, PINE cohort, data collection; Hazel Forbes, University of Aberdeen, UK, Co‐Investigator, PINE cohort, data collection; Valerie Angus, University of Aberdeen, UK, Co‐Investigator, PINE cohort, data management; Alasdair Finlayson, University of Aberdeen, UK, Co‐Investigator, PINE cohort, data management; David Dawson, University of Aberdeen, UK, Co‐Investigator, PINE cohort, data management; Katie Wilde, University of Aberdeen, UK, Co‐Investigator, PINE cohort, data management; David Ritchie, University of Aberdeen, UK, Co‐Investigator, PINE cohort, data management; Artur Wozniak, University of Aberdeen, UK, Co‐Investigator, PINE cohort, data management; Adrian Martin, University of Aberdeen, UK, Co‐Investigator, PINE cohort, data management.
