# Supplementary material for: Prognosis in Parkinson's Disease: An Individual Patient Data Meta‐Analysis of Six European Incidence Cohorts
Source: Mov Disord. 2026 Apr 11;41(7):1798–811. doi: 10.1002/mds.70303 (PMC13387983; doi:10.1002/mds.70303)
Supplement: Supplementary file 1 — Appendix S1. Medline systematic literature search strategy. Table S1. Characteristics of incidence cohorts included in the Parkinson's Incidence Cohorts Collaboration. Table S2. Participants' baseline characteristics. Table S3. Prognostic factors for key outcomes in the Parkinson's Incidence Cohorts Collaboration (univariable and multivariable analysis). Table S4. Probabilities of developing four key outcomes in Parkinson's disease at 5 and 10 years from diagnosis, by age stratum. Table S5. Results of systematic search of published literature – previous studies reporting longitudinal outcomes (postural instability, functional dependency, dementia, and mortality). Table S6. Results of systematic search of published literature – previous studies reporting prognostic factors for functional dependency, dementia, and mortality. Table S7. Previous Parkinson's Incidence Cohorts Collaboration publications on dependency, postural instability dementia, and mortality. Table S8. Association between sex and mortality in inception studies in Parkinson's disease and Supplemental References. [file MDS-41-1798-s001.docx]

**Supplemental material**

**Prognosis in Parkinson’s disease: an individual-patient-data meta-analysis of six European incidence cohorts**

Angus D Macleod PhD,^1^ David J McLernon PhD,^1^ Marta Camacho PhD,^2^ Caroline H Williams-Gray PhD,^2^ Rachael A Lawson PhD,^3^ Alison J Yarnall PhD,^3^ David Backstrom PhD,^4^ Lars Forsgren PhD,^4^ Jodi Maple-Grødem PhD,^5^ Guido Alves PhD,^5^ Ole-Bjørn Tysnes PhD,^6^ & Carl E Counsell MD^1^ on behalf of the Parkinson’s Incidence Cohorts Collaboration

**Contents:**

1. Supplemental Appendix 1: Medline systematic literature search strategyPage 2
2. Supplemental Table 1 Characteristics of incidence cohorts included in the Parkinson’s Incidence Cohorts CollaborationPage 3
3. Supplemental Table 2: Participants’ baseline characteristicsPage 4
4. Supplemental Table 3: Prognostic factors for key outcomes in the Parkinson’s Incidence Cohorts Collaboration (univariable and multivariable analysis)Page 6
5. Supplemental Table 4: Probabilities of developing four key outcomes in Parkinson’s disease at five years and ten years from diagnosis, by age stratum (45-64, 65-74. 75-84. ≥85)Page 8
6. Supplemental Table 5: Results of systematic search of published literature – previous studies reporting longitudinal outcomes (postural instability, functional dependency, dementia, and mortality)Page 9
7. Supplemental Table 6: Results of systematic search of published literature– previous studies reporting prognostic factors for functional dependency, dementia, and mortalityPage 18
8. Supplemental Table 7: Previous PICC publications on dependency, postural instability dementia, and mortalityPage 26
9. Supplemental Table 8: Association between sex and mortality in inception studies in Parkinson’s diseasePage 27
10. Supplemental ReferencesPage 28

**Supplemental Appendix 1: Medline systematic literature search strategy**

| 1. | exp Parkinson Disease/ep, mo, sn [Epidemiology, Mortality, Statistics & Numerical Data] |
| --- | --- |
| 2. | (longitudinal or cohort or follow-up or followup or followed up or disease progression or prognos$).tw. |
| 3. | exp Cohort Studies/ |
| 4. | mortality/ or survival rate/ |
| 5. | (mortality or death or dementia or dependenc* or independenc* or instab$).tw. |
| 6. | 1 and (2 or 3) and (4 or 5) |
| 7. | exp animals/ not humans.sh. |
| 8. | 6 not 7 |

**Supplemental Table 1:** **Characteristics of incidence cohorts included in the Parkinson’s Incidence Cohorts Collaboration**

| **Study** | **CamPaIGN** | **ICICLE-PD** | **NYPUM** | **ParkWest** | **PICNICS** | **PINE** | **Pooled cohort** |
| --- | --- | --- | --- | --- | --- | --- | --- |
| Location | Cambridgeshire, UK | Newcastle/Gateshead, UK | Umeå, Sweden | Western Norway | Cambridgeshire, UK | Aberdeen, UK |  |
| Recruitment period | 2000-2 | 2009-11 | 2004-9 | 2004-6 | 2008-10 | 2002-4;06-9 | 2000-2011 |
| Consent to follow-up amongst incident population | 79% | 48% | 94% | 79% | 74% | 94% | 78% |
| Number in cohort | 140 | 82 | 144 | 191 | 125 | 201 | 883 |
| Median follow-up in years | 7.6 | 6.0 | 8.1 | 7.0 | 10.9 | 7.7 | 7.1 |
| Max follow- up duration in years | 10.2 | 8.5 | 12.0 | 7.9 | 12.0 | 12.0 | 12.0 |

**Supplemental Table 2: Participants’ baseline characteristics**

| **Baseline Characteristic** | **CamPaIGN**  N=140 | **ICICLE-PD**  N=82 | **NYPUM**  N=144 | **ParkWest**  N=191 | **PICNICS**  N=125 | **PINE**  N=201 | **Pooled cohort**  N=883 |
| --- | --- | --- | --- | --- | --- | --- | --- |
| Median age at motor symptom onset (IQR)  Missing, N (%) | 69.5 (62.2–75.1)  1 (1) | 65.8 (59.7–72.7)  7 (9) | 70.9 (61.6–76.2)  0 (0) | 65.8 (60.2–72.3)  0 (0) | 67.4 (60.7–73.9)  1 (1) | 72.0 (65.0–78.0)  0 (0) | 69.2 (61.2–75.0)  5  9 (1) |
| Median age at diagnosis (IQR)  Missing, N (%) | 71.8 (64.4–76.9)  0 (0) | 68.1 (60.4–74.2)  0 (0) | 72.4 (63.7–78.3)  0 (0) | 68.4 (62.4–74.8)  0 (0) | 68.7 (62.6–75.0)  0 (0) | 73.0 (68.0–79.0)  0 (0) | 71.0 (63.4–77.0)  0 (0) |
| Age at baseline study assessment  Age <50, N (%)  Age 50-59, N (%)  Age 60-69, N (%)  Age 70-79, N (%)  Age 80+, N (%)  Median (IQR)  Missing, N (%) | 4 (3)  14 (10)  39 (28)  57 (41)  26 (19)  72.2 (64.6–77.1  0 (0)) | 8 (10)  7 (9)  32 (39)  26 (32)  9 (11)  68.3 (60.9–74.4)  0 (0) | 3 (2)  20 (14)  35 (24)  62 (43)  24 (17)  72.4 (63.7–78.3)  0 (0) | 6 (3)  30 (16)  72 (38)  64 (34)  19 (10)  68.5 (62.5–75.0)  0 (0) | 3 (2)  16 (13)  45 (36)  50 (40)  11 (9)  69.3 (62.8–75.4)  0 (0) | 9 (4)  17 (8)  31 (15)  93 (46)  51 (25)  73.8 (68.3–80.0)  0 (0) | 33 (4)  104 (12)  254 (29)  352 (40)  140 (16)  71.4 (63.6–77.3)  0 (0) |
| Median time in years from motor onset to baseline visit (IQR) | 1.7 (1.0–3.0) | 0.7 (0.4–1.2) | 1.3 (0.9–2.5) | 1.6 (1.1–3.1) | 1.5 (1.0–1.9) | 1.2 (0.8–2.1) | 1.4 (0.9–2.2) |
| Male, N (%)  Missing, N (%) | 78 (56)  0 (0) | 53 (65)  0 (0) | 86 (60)  0 (0) | 117 (61)  0 (0) | 83 (66)  0 (0) | 123 (61)  0 (0) | 540 (61)  0 (0) |
| White ethnicity, N (%)  Missing, N (%) | 138 (99)  0 (0) | 78 (95)  0 (0) | 144 (100)  0 (0) | 191 (100)  0 (0) | 124 (99)  0 (0) | 200 (100)  0 (0) | 875 (99)  0 (0) |
| N with family history of PD^a^  Missing, N (%) | 18 (13)  5 (4) | 9 (12)  4(5) | 21 (15)  0 (0) | 23 (12)  1 (1) | 7 (6)  3 (2) | 25 (12)  0 (0) | 103 (12)  13 (1) |
| Median years of education (IQR)  Missing, N (%) | 10 (9–12)  5(4) | 11 (10–13)  0 (0) | 8 (7–12)  11 (8) | 11 (9–13)  0 (0) | 11 (10–13)  0 (0) | 11 (10–14)  4 (2) | 11 (9–13)  20 (2) |
| Smoking history  N current smoker (%)  N ex-smoker (%)  Missing, N (%) | 5 (4)  76 (54)  0 (0) | 6 (7)  37 (46)  1 (1) | 4 (3)  44 (31)  2 (1) | 18 (9)  77 (40)  0 (0) | 4 (3)  33 (28)  6 (5) | 12 (6)  79 (40)  1 (1) | 49 (6)  347 (40)  10 (1) |
| N with hallucinations (%)  No  Yes  Missing | 135 (97)  4 (3)  1 (1) | 67 (82)  15 (18)  0 (0) | 140 (99)  2 (1)  2 (1) | 189 (99)  2 (1)  0 (0) | 121 (97)  4 (3)  0 (0) | 158 (93)  11 (7)  0 (0) | 743 (97)  23 (3)  117 (13) |
| Cognitive symptoms^b^  None  Present, not impairing functioning  Present, impairing functioning  Missing | 60 (43)  59 (42)  20 (14)  1 (1) | NA  82 (100) | 110 (77)  32 (23)  0 (0)  2 (1) | 129 (67)  54 (28)  8 (4)  0 (0) | 83 (66)  39 (31)  3 (2)  0 (0) | 103 (61)  55 (33)  10 (6)  33 (16) | 485 (63)  239 (31)  41 (5)  118 (13) |
| H&Y stage, N (%)  1/1.5  2/2.5  3  4  Mean H&Y stage (SD)  Missing, N (%) | 42 (30)  82 (59)  14 (10)  2 (1)  2.0 (0.7)  0 (0) | 19 (23)  42 (51)  20 (24)  1 (1)  2.0 (0.7)  0 (0) | 19 (13)  89 (62)  29 (20)  7 (5)  2.3 (0.7)  2 (1) | 78 (41)  94 (49)  16 (8)  3 (2)  1.9 (0.6)  0 (0) | 61 (49)  53 (42)  11 (9)  0 (0)  1.6 (0.6)  0 (0) | 48 (24)  101 (50)  39 (19)  13 (6)  2.3 (0.8)  3 (1) | 267 (30)  461 (52)  129 (15)  26 (3)  2.0 (0.8)  5 (1) |
| Mean original UPDRS part 3 (SD)  Missing, N (%) | 26.4 (12.5)  1 (1) | NA  82 (100) | 27.4 (11.7)  0 (0) | 23.4 (11.2)  0 (0) | NA  125 (100) | 25.0 (11.5)  1 (1) | 25.4 (11.7)  209 (24) |
| Mean MDS-UPDRS part 3 (SD)^c^  Missing, N (%) | 33.8 (14.8)  1 (1) | 27.5 (12.3)  1 (1) | 34.9 (13.9)  0 (0) | 30.3 (13.3)  0 (0) | 30.2 (11.6)  1 (1) | 32.1 (13.6)  1 (1) | 31.8 (13.6)  3 (0) |
| UPDRS axial score^d^  Missing, N (%) | 3 (1-5.5)  1 (1) | 3 (2-5)  1 (1) | 3 (1-4)  0 (0) | 3 (1-4)  0 (0) | 2 (1-4)  0 (0) | 3 (2-5)  1 (1) | 3 (1-5)  3 (0) |
| Mean S&E (SD)  Missing, N (%) | 85 (60-100)  0 (0) | NA  82 (100) | 90 (85-90)  2 (1) | 90 (80-90)  0 (0) | NA  125 (100) | 90 (80-95)  0 (0) | 90 (80-90)  209 (24) |
| MMSE, N (%)  30  29  27/28  <27  Median MMSE (IQR)  Missing, N (%) | 25 (18)  34 (24)  58 (41)  23 (16)  28 (27-29)  0 (0) | 22 (27)  26 (32)  27 (33)  7 (9)  29 (28-30)  0 (0) | 47 (33)  29 (20)  43 (30)  15 (10)  29 (28-30)  10 (7) | 42 (22)  53 (28)  54 (28)  42 (22)  28 (27-29)  0 (0) | 41 (33)  45 (36)  30 (24)  9 (7)  29 (28-30)  0 (0) | 49 (24)  61 (30)  51 (25)  25 (14)  29 (28–29)  15 (7) | 226 (26)  248 (28)  263 (30)  121 (14)  29 (27–30)  25 (3) |
| APOE ε4 carrier  Missing | 33 (27)  17 (12) | 23 (32)  9 (11) | 33 (25)  11 (8) | 61 (32)  2 (1) | 26 (23)  13 (10) | 46 (32)  56 (28) | 222 (29)  108 (12) |
| MAPT H1/H1 haplotype  Missing | 82 (65)  14 (10) | 56 (75)  7 (9) | 98 (74)  11 (8) | 143 (76)  3 (2) | 81 (66)  3 (2) | 100 (69)  56 (28) | 560 (71)  94 (11) |
| Any GBA mutation  Missing | 20 (18)  28 (20) | 3 (4)  12 (15) | 19 (14)  12 (8) | 21 (11)  2 (1) | 8 (6)  1 (1) | 11 (9)  84 (42) | 82 (9)  139 (16) |

^a^In a first-degree relative; ^b^derived from UPDRS/MDS-UPDRS part 1; ^c^Converted from original UPDRS where necessary using the formula from Goetz et al. ^d^Sum of axial items in part 3 MDS-UPDRS or original UPDRS (sum of arising from chair, posture, gait, and postural instability items). Abbreviations: H&Y=Hoehn & Yahr stage; IQR=inter-quartile range; MDS-UPDRS=Movement Disorder Society revision of UPDRS; MMSE=mini-mental state examination; N=number; NA=not available; S&E= Schwab & England scale; SD=standard deviation; UPDRS=Unified Parkinson's disease rating scale.

**Supplemental Table 3: Prognostic factors for key outcomes in the Parkinson’s Incidence Cohorts Collaboration (univariable and multivariable analysis)**

| **Baseline prognostic factor** | **Outcome** | | | | | | | | | | | | | | | |
| --- | --- | --- | --- | --- | --- | --- | --- | --- | --- | --- | --- | --- | --- | --- | --- | --- |
|  | **Time to sustained postural instability**  (Hoehn & Yahr stage 3)  N=741^a^ | | | | **Time to sustained dependency**  N=742^b^ | | | | **Time to dementia**  N=853^c^ | | | | **Time to death**  N=880^d^ | | | |
|  | Univariable | | Multivariable | | Univariable | | Multivariable | | Univariable | | Multivariable | | Univariable | | Multivariable | |
|  | HR  (95% CI) | P-value | HR  (95% CI) | P-value | HR  (95% CI) | P-value | HR  (95% CI) | P-value | HR  (95% CI) | P-value | HR  (95% CI) | P-value | HR  (95% CI) | P-value | HR  (95% CI) | P-value |
| **Age at baseline**  **(HR for 10-year increase)** | 2.65  (2.30–3.04) | <0.001 | 2.61  (2.23–3.05) | <0.001 | 2.27  (1.99–2.59) | <0.001 | 2.05  (1.77–2.37) | <0.001 | 2.29  (1.98–2.66) | <0.001 | 1.93  (1.63–2.28) | <0.001 | 2.88  (2.52–3.28) | <0.001 | 2.44  (2.11–2.82) | <0.001 |
| **Female vs male sex** | 1.07  (0.87–1.32) | 0.53 |  |  | 0.83  (0.67–1.02) | 0.08 | 0.94  (0.75–1.18) | 0.60 | 0.93  (0.73–1.18) | 0.54 | 1.19  (0.92–1.54) | 0.18 | 0.70  (0.57–0.86) | 0.001 | 0.74  (0.59–0.91) | 0.005 |
| **Female vs male**  **Up to 6 years**  **Year 6 onwards** |  |  | 1.05  (0.81–1.36)  2.82  (1.81–4.39) | 0.72  <0.001 |  |  |  |  |  |  |  |  |  |  |  |  |
| **Years of education** | 0.98  (0.95–1.01) | 0.24 | 1.05  (1.01–1.08) | 0.005 | 0.96  (0.93–0.99) | 0.01 | 1.03  (1.00–1.06) | 0.08 | 0.93  (0.89–0.96) | <0.001 | 1.02  (0.98–1.06) | 0.31 | 0.95  (0.92–0.98) | 0.001 | 1.03  (0.99–1.06) | 0.11 |
| **Current vs never smoker** | 1.08  (0.68–1.72) | 0.73 | 1.34  (0.83–2.17) | 0.24 | 0.98  (0.61–1.58) | 0.95 | 1.19  (0.73–1.94) | 0.49 | 0.96  (0.55–1.66) | 0.88 | 0.97  (0.55–1.71) | 0.92 | 1.17  (0.74–1.85) | 0.51 | 1.38  (0.86–2.22) | 0.18 |
| **Ex vs never smoker** | 1.16  (0.94–1.45) | 0.17 | 0.99  (0.78–1.26) | 0.95 | 1.27  (1.02–1.57) | 0.03 | 1.01  (0.80–1.28) | 0.91 | 1.16  (0.91–1.48) | 0.22 | 0.87 (0.66–1.14) | 0.30 | 1.48  (1.21–1.81) | 0.51 | 1.21  (0.98–1.50) | 0.08 |
| **Presence of hallucinations** | 2.35  (1.48–3.74) | 0.001 | 1.44  (0.80–2.60) | 0.22 | 2.99  (1.86–4.81) | <0.001 | 2.14  (1.13–4.07) | <0.02 | 2.62  (1.62–4.23) | <0.001 | 2.58  (1.45–4.29) | 0.001 | 2.33  (1.56–3.47) | 0.001 | 1.54  (1.01–2.34) | 0.04 |
| **Cognitive symptoms not impairing functioning vs no cognitive symptoms** | 1.81  (1.44–2.29) | <0.001 | 1.23  (0.96–1.58) | 0.10 | 2.13  (1.70–2.68) | <0.001 | 1.38  (1.08–1.75) | 0.01 | 2.23  (1.71–2.89) | <0.001 | 1.73  (1.30–2.31) | <0.001 | 1.72  (1.65–1.80) | <0.001 | 1.06  (0.84–1.34) | 0.65 |
| **Cognitive symptoms impairing functioning vs no cognitive symptoms** | 5.47  (3.38–8.84) | <0.001 | 2.59  (1.52–4.40) | <0.001 | 5.42  (3.31–8.88) | <0.001 | 1.78  (0.98–3.21) | 0.06 | 4.99  (3.20–7.78) | <0.001 | 2.44  (1.39–4.29) | 0.002 | 3.77  (3.48-4.08) | <0.001 | 1.39  (0.91–2.12) | 0.13 |
| **Hoehn & Yahr stage** | 1.66  (1.41–1.95) | <0.001 | 1.14  (0.92–1.41) | 0.24 | 1.79  (1.53–2.10) | <0.001 | 1.01  (0.82–1.26) | 0.90 | 2.09  (1.79–2.44) | <0.001 | 1.12  (0.91–1.38) | 0.30 | 2.03  (1.79–2.30) | <0.001 | 1.21  (1.02–1.43) | 0.03 |
| **MDS-UPDRS part III**  **(HR for 10-point increase)** | 1.42  (1.31–1.54) | <0.001 | 1.36  (1.22–1.51) | <0.001 | 1.42  (1.31–1.53) | <0.001 | 1.30  (1.18–1.45) | <0.001 | 1.47  (1.36–1.60) | <0.001 | 1.27  (1.13–1.43) | <0.001 | 1.49  (1.39–1.60) | <0.001 | 1.22  (1.11–1.34) | <0.001 |
| **Axial severity score^e^** | 1.39  (1.32–1.45) | <0.001 | 1.30  (1.24–1.38) | <0.001 | 1.30  (1.25–1.35) | <0.001 | 1.18  (1.12–1.25) | <0.001 | 1.23  (1.19–1.28) | <0.001 | 1.11  (1.04–1.18) | 0.003 | 1.22  (1.18–1.25) | <0.001 | 1.05  (1.00–1.10) | 0.045 |
| **MMSE: 29 vs 30** | 1.75  (1.29–2.36) | <0.001 | 1.59  (1.17–2.15) | 0.003 | 1.59  (1.17–2.14) | 0.003 | 1.36  (1.01–1.85) | 0.05 | 01.43  (0.99–2.08) | <0.06 | 1.34  (0.91–1.97) | 0.14 | 1.33  (0.99–1.79) | 0.06 | 1.08  (0.81–1.47) | 0.58 |
| **MMSE: 27/28 vs 30** | 2.43  (1.81–3.26) | <0.001 | 1.42  (1.04–1.92) | 0.03 | 2.56  (1.92–3.42) | <0.001 | 1.63  (1.20–2.21) | 0.002 | 2.93  (2.08–4.13) | <0.001 | 2.09  (1.44–3.02) | <0.001 | 2.38  (1.81–3.14) | <0.001 | 1.43  (1.07–1.90) | 0.02 |
| **MMSE: <27 vs 30** | 2.71  (1.86–3.95) | <0.001 | 1.36  (0.90–2.05) | 0.14 | 3.41  (2.36–4.90) | <0.001 | 2.17  (1.44–3.26) | <0.001 | 5.24  (3.56–7.71) | <0.001 | 3.32  (2.12–5.20) | <0.001 | 3.63  (2.64–5.00) | <0.001 | 1.55  (1.09–2.22) | 0.02 |
| **APOE ε4 carrier** | 0.93  (0.73–1.20) | 0.58 | 1.14  (0.89–1.46) | 0.31 | 0.80  (0.62–1.03) | 0.08 | 0.98  (0.75–1.28) | 0.86 | 1.57  (1.21–2.02) | 0.001 | 2.14  (1.59–2.89) | <0.001 | 1.21  (0.96–1.52) | 0.11 | 1.42  (1.12–1.80) | 0.004 |
| **MAPT H1/H1 haplotype** | 0.92  (0.72–1.17) | 0.49 | 1.05  (0.82–1.35) | 0.70 | 0.82  (0.65–1.04) | 0.11 | 0.86  (0.68–1.10) | 0.23 | 1.02  (0.77–1.34) | 0.89 | 1.10  (0.83–1.44) | 0.51 | 0.96  (0.77–1.21) | 0.74 | 1.08  (0.86–1.36) | 0.50 |
| **Any GBA mutation** | 1.27  (0.91–1.75) | 0.16 | 1.72  (1.23–2.41) | 0.002 | 1.30  (0.94–1.80) | 0.11 | 1.79  (1.26–2.52) | 0.001 | 1.52  (1.08–2.14) | 0.02 | 2.18  (1.50–3.16) | <0.001 | 0.85  (0.59–1.22) | 0.37 | 1.02  (0.70–1.50) | 0.91 |

^a^92 participants had sustained postural instability from baseline and were excluded, 50 participants had no follow-up data for this outcome (14 due to death before the first follow-up visit and 36 due to loss to follow-up. ^b^3 participants had no data available for this variable, 99 participants had sustained dependency from baseline, and 39 participants had no follow-up data for this outcome (12 due to death before first follow-up visit and 27 due to loss to follow-up). ^c^5 participants had dementia at baseline and were excluded; 25 participants had missing dementia data due to loss to follow-up for this outcome. ^d^3 participants had missing data for death due to loss to follow-up. ^e^Sum of axial items in part 3 MDS-UPDRS or original UPDRS (sum of arising from chair, posture, gait, and postural instability items). APOE=apolipoprotein E; CI=confidence interval; GBA=glucocerebrosidase; HR=hazards ratio; MAPT=microtubule-associated protein tau; MDS-UPDRS=Movement Disorders Society revision of the Unified Parkinson's Disease Rating Scale; MMSE=mini-mental state examination.

**Supplemental Table 4: Probabilities of developing four key outcomes in Parkinson’s disease at five years and ten years from diagnosis, by age stratum (45-54, 55-64, 65-74. 75-84. ≥85)**

| **Age stratum** | **Postural instability^a^ (men and women)** | **Functional dependency^b^ (men and women)** | **Dementia (men and women)** | **Mortality in men** | **Mortality in women** |
| --- | --- | --- | --- | --- | --- |
|  | ***Percentage with outcome at five years from diagnosis*** | | | | |
| **Age 45-54** | 6 | 8 | 2 | 3 | 0 |
| **Age 55-64** | 22 | 15 | 9 | 6 | 4 |
| **Age 65-74** | 35 | 35 | 28 | 17 | 9 |
| **Age 75-84** | 60 | 61 | 43 | 31 | 32 |
| **Age ≥85** | 74 | 86 | 53 | 78 | NR |
|  | ***Percentage with outcome at ten years from diagnosis*** | | | | |
| **Age 45-54** | 24 | 39 | 10 | 15 | 7 |
| **Age 55-64** | 52 | 52 | 33 | 26 | 17 |
| **Age 65-74** | 77 | 80 | 54 | 58 | 40 |
| **Age 75-84** | 94 | 91 | 71 | 87 | 79 |
| **Age ≥85** | NR | NR | NR | NR | NR |

^a^In those without postural instability at baseline. ^b^In those independent at baseline. NR: estimated not reported due to small numbers in these strata.

**Supplemental Table 5: Results of systematic search of published literature – previous longitudinal studies reporting (postural instability, functional dependency, dementia, and mortality)**

| **Study** | **Country** | **Inception study?** | **Population/hospital recruitment** | **N with outcome/ total N** | **Follow-up in years** | **Measure(s)** | **Definition** | **Value** | **Notes** |
| --- | --- | --- | --- | --- | --- | --- | --- | --- | --- |
| **Dependency outcomes over time** | | | | | | | | | |
| Auyeung 2012^1^ | Hong Kong | Inception | Hospital | 56/110 | Mean 11.3 | Risk in surviving patients | S&E <80% | 46% |  |
| Bjornestad 2016^2^ | Norway | Inception | Population | 65/159 | 5 | Cumulative incidence | Based on interview | 40.9 | Denominator is those independent at baseline.  ParkWest study, included in PICC. |
| Hely 2008^3^ | Australia | Inception | Hospital | 28/57 at 10 y  26/40 at 15y  29/30 at 20y | Up to 20 | Risk in surviving patients | H&Y stage >3 | 49% at 10 y  65% at 15 y  97% at 20 y |  |
| Jasinska-Myga 2012^4^ | USA | Non-inception | Hospital | NS/491 | Median 4, up to 10 | Risk in surviving patients | Based on interview | 16% at 4 y  27% at 6 y  39% at 8 y  56% at 10 y | Overlap with Konno 2018 |
| Konno 2018^5^ | USA | Non-inception | Hospital | 57/648 | Range 0-22.9, median 3.7 | Risk | Help with basic ADLs or in NH | 88% | Retrospective cohort. Overlap with Jasinska-Myga 2012 |
| Macleod 2016^6^ | UK | Inception | Population | NS/164 | Up to 12 | Median time to dependency  KM probability | S&E <80% | 5.5 y  95% at 10 y | Denominator is those independent at baseline. PINE study, included in PICC. |
| Santos Garcia 2021^7^ | Spain | Non-inception | Hospital | NS/507 | 2 | Risk in surviving patients | S&E<80% | 8.7% dep at BL, 16.8% (of available patients) at 2 y | 26% dropped out of study by year 2. |
| Scigliano 1990^8^ | Italy | Non-inception | Hospital | 87/155 | Mean 4.4 | Risk in surviving patients | Not defined | 56% | 51% of cohort didn’t respond to question about dependency |
| **Postural instability outcomes over time** | | | | | | | | | |
| Hiorth 2017^9^5 | Norway | Inception | Population | 88/153 | 7 | Cumulative incidence | Falls recorded at Clinical interview | 57.5 | In those without falls at baseline. ParkWest study, included in PICC. |
| Konno 2018^5^ | USA | Non-inception | Hospital | 262/718 | Range 0-22.9, median 3.7 | Falling within 5 years of onset | Unclear | 36.5 |  |
| Ou 2021^10^ | China | Inception | Hospital | NS/379 | Up to 14 | Median time to HY3 | HY3 | 8.3 y | Subset of patient with blood tests available. Immortal time at start of study |
| Sato 2006^11^ | Japan | Inception | Hospital | NS/1178 | Up to 14 | Risk (KM probability) | HY3 | 30.2% at 5y, 57.2% at 10 y, 83.5% at 15 y |  |
| Williams-Gray 2013^12^ | UK | Inception | Population | 81/142 | 10 | Median time | HY3 | 4.7 y | CamPaIGN study, included in PICC. |
| **Dementia outcomes over time** | | | | | | | | | |
| Anang 2014^13^ | Canada | Non-inception | Hospital | 27/80 | 4.4 | Risk in surviving patients | Level 2 MDS criteria | 34% |  |
| Anang 2017^14^ | Canada/ Japan | Non-inception | Hospital | 30/134 | 4.2 | Risk in surviving patients | MDS criteria | 22% |  |
| Åström 2022^15^ | Sweden | Non inception | Unclear | 651/1362 | Mean 9.4 | KM probability  Median time | ICD-10 coding | Approx 16% at 10 y  Median about 20 y | Registry study. Partly retrospective. Bias from measuring survival to dementia from diagnosis when many cases recruited long after diagnosis (immortal time bias).  More data in KM plot. |
| Bäckström 2022^16^ | Sweden | Inception | Population | 64/143 | 10 | Incidence rate  Cumulative probability | MDS criteria | 56.2 (95%CI 44.2–71.2) per 1000 py  54% | NYPUM study, included in PICC. |
| Bakeberg 2020^17^ | Australia | Non-inception | Hospital | NS/127 | 3.0 | Risk (KM probability) | ACE-R <83 | 20% at 4 y | Data from KM plot |
| Biggins 1992^18^ | UK | Non-inception | Hospital | 10/82 | Up to 4.5 | Risk (KM probability) | DSM-III-R | 19% |  |
| Buter 2008^19^ | Norway | Non-inception | Population | 140/233 | 12 | Cumulative incidence | DSM-III-R | 45% at 4 y  60% at 12 y | Norway prevalence study |
| Campbell 2020^20^ | USA | Non-inception | Hospital + Population | 48/162 | NS, up to 10 | Cumulative incidence | CDR ≥ 1 | 28.4% | KM plot |
| Counsell 2022^21^ | UK | Inception | Population | 93/197 | Median 9.5 | Incidence rate  Median time  KM probability | DSM-IV | 7.4 cases per 100 py (95% CI 6.1–9.1)  8.5 y  58.1% at 10 y | PINE study, included in PICC. |
| De Lau 2005^22^ | Netherlands | Non-inception | Population | 21/139 | Mean 6.9 | Cumulative incidence | DSM-III-R | 15.1% | Combined incidence + prevalence without dementia at baseline. |
| Hely 2008^3^ | Australia | Inception | Hospital | 97/136 | Up to 20 | Risk (KM probability) | Clinical Dementia Rating >=1 or neuropsychology assessment. | 83% at 20 y  Median 15 y but see note | Note: most diagnoses of dementia came at 10y and 15y so true median time almost certainly shorter than 15 |
| Hughes 2000^23^ | UK | Non-inception | Hospital | 17/90 | Up to 10 | Median (KM) | DSM-III-R | 10 y |  |
| Janvin 2005^24^ | Norway | Non-inception | Population | 25/60 | 4 | Risk in surviving pts | DSM-III-R | 42% | Same study as Buter but confined to those without dementia at baseline |
| Keener 2018^25^ | USA | Inception | Population | 34/224 | 5.7 | Risk in surviving patients | MMSE <24 | 15% |  |
| Konno 2018^5^ | USA | Non-inception | Hospital | 68/1003 | Range 0-22.9, median 3.7 | Risk in surviving patients | NS | 6% at 5 y  9.8% at end of FU |  |
| Kwon 2014^26^ | Korea | Inception | Hospital | 18/80 | 2.2 | Risk in all patients | MDS criteria | 23 |  |
| Levy 2002^27^ | USA | Non-inception | Population | 52/180 | Mean 3.6 | Risk in all patients | DSM-III-R | 29% | Denominator slightly different in Levy 2000 (173 vs 180) |
| Liu 2019^28^ | Taiwan | Inception | Population | 1836/5932 | Up to 11 | Risk (KM probability) | ICD-9 coding | 28% (KM) at 5 y.  Median not reached by 11 y | Diagnoses based on medication codes. Validation study: 97.6 sensitivity, 92.3% specificity.  More data in KM plot |
| Malek 2016^29^ | UK | Inception | Hospital | 103/1424 | 3.7 | Risk in patients followed-up | MDS criteria | 7% | Also data in Real 2023 with longer follow-up |
| Nicoletti 2021^30^ | Italy | Non-inception | Hospital | 18/139 | 2 | Risk in all patients | MDS criteria | 13% |  |
| Real 2023^31^ | Multiple | Both | Hospital | 265/3923 | Mean 3.6 | Risk in all patients | Varies | 6.8% | One of included cohorts also reported in Malek 2016 |
| Roos 1996^32^ | Netherlands | Non-inception | Hospital | 90/345 | Up to 30 | Cumulative incidence | MMSE <25 | 26% | Partly retrospective |
| Stern 1993^33^ | US | Non-inception | Hospital | 74/250 | 2.5 | Risk in surviving patients | DSM III | 29.6% |  |
| Williams-Gray 2013^12^ | UK | Inception | Population | 41/142 | 10 | Incidence rate  KM probability | DSM-IV | 54.7 per 1000 py (95%CI 35.4–74.1)  46% at 10 y | CamPaIGN study, included in PICC. |
| Wu 2016^34^ | Taiwan | Inception | Population | 169/1213 | Mean 6.3 | Incidence rate | ICD-9 coding | 24.2/1000py | Population study |
| **Mortality outcomes over time** | | | | | | | | | |
| Auyeung 2012^1^ | Hong Kong | Inception | Hospital | 50/171 | 11.3 | SMR; Survival | NA | 1.1 (0.8–1.5)  Survival 97.1% at 5y, 83.7% at 10 y, median >16 y |  |
| Bäckström 2018^35^ | Sweden | Inception | Population | 77/143 | Up to 13.5 | SMR  Median survival | NA | 1.58 (1.25–1.98)  9.6 y | NYPUM study, included in PICC. |
| Ben-shlomo 1995^36^ | UK | Non-inception | Population | 195/220 | Median 17.2 | Median survival | NA | 8.28 y from recruitment |  |
| Berger 2000^37^ | Europe | Non-inception | Population | NS/139 | 3-10 | HR (vs controls) | NA | 2.32 (1.80–300) |  |
| Bjornestad 2017^38^ | Norway | Inception | Population | 34/185 | 7 | Cumulative incidence | NA | 34% | ParkWest study, included in PICC, |
| Bugalho 2019^39^ | Portugal | Non-inception | Hospital | 22/130 | Up to 4 | Mortality Risk | NA | 16.9% | Adjusted for age, gender and disease duration |
| Campbell 2020^20^ | USA | Non-inception | Hospital + Population | 46/162 | Up to 10 | KM survival | NA | 90% at 5 y, 40% at 10 y | Estimated from KM plot |
| Chia 1992^40^ | Taiwan | Non inception | Hospital | 46/215 | Mean 5.2 | Mortality risk | NA | 21.4% |  |
| Chillag-Talmor 2013^41^ | Israel | Inception | Population | NS/4848 | Mean 4.0, up to 10 | Cumulative incidence | NA | 26% | Incident patients identified by drug claims. Note algorithm sensitive for PD but not specific – 82% of parkinsonism cases falsely identified as PD. |
| D’Amelio 2006^42^ | Italy | Non-inception | Population | 46/58 | Mean 7.7 | Median | NA | 6.8 y from recruitment |  |
| Das 2010^43^ | India | Non-inception | Population | 13/37 | 6 | Median  SMR | NA | 13.5 y from onset  3.79 (2.01–6.48) |  |
| de Lau 2005^22^ | Netherlands | Non-inception | Population | 90/164 | Mean 6.9 | Median  HR vs controls | NA | 9.1 y from dx (7.4–10.9)  1.83 (1.47–2.26) |  |
| de Lau 2014^44^ | Netherlands | Non-inception | Hospital | 79/358 | Mean 4.32 | Mortality risk | NA | 13.7 | Excluding 56 lost to FU |
| Diamond 1987^45^ | USA | Non-inception | Hospital | 171/371 | Mean 10.3 | SMR | NA | 1.95 (1.78–2.12) |  |
| Driver 2008^46^ | USA | Inception | Other | 200/560 | Mean 5.8 | Median; HR | NA | 11.8 y from dx; HRs 2.05 (1.60–2.56) | US physicians study. |
| Duarte 2013^47^ | Spain | Inception | Population | 69/273 | Up to 30 | Median (KM) | NA | 11 y | KM plot. Medians also by age group |
| Elbaz 2003^48^ | US | Inception | Population | 79/196 | Median 7.2 | Median; KM survival | NA | Median 10.3 from dx; 79% at 5 y; 53% at 10 y |  |
| Fall 2003^49^ | Sweden | Non-inception | Population | 121/170 | 9 | Median | NA | 6.3 |  |
| Fielding 2016^50^ | UK | Inception | Population | 73/199 | Up to 1 | Median | NA | 7.8 y | More data in KM plot. PINE study, included in PICC. |
| Forsaa 2010^51^ | Norway | Non-inception | Population | 211/220 | 10 | SMR | NA | 1.52 (1.29–1.79) |  |
| Gonzalez 2022^52^ | Norway | Inception | Population | 65/190 | 10 | KM probability | NA | 37.5% at 10 y | More data in KM plot. ParkWest study, included in PICC. |
| Guttman 2001^53^ | Canada | Non-inception | Population | 7779/15165 | 6 | Median | NA | 6 y |  |
| Hely 2008^3^ | Australia | Inception | Hospital | 100/130 | 20 | Median; Survival | NA | 12.2; 97.8%; at 5 y; 61.5% at 10 y; 23.1% at 20 y |  |
| Hobson 2018^54^ | UK | Non-inception | Population | 158/166 | Up to 18 | Median (KM) | NA | 3.6 y | KM |
| Hoehn 1967^55^ | US | Inception | Hospital | 91/241 | Mean about 7 | SMR | NA | 2.9 (2.33–3.56) |  |
| Hoogland 2019^56^ | Netherlands | Inception | Hospital | 85/129 | Average 10.3 | Median | NA | 11.8 y | Excluded >85s (and some other exclusions which could lead to bias) |
| Hughes 2004^57^ | UK | Non-inception | Hospital | 50/89 | Up to 11 | Median  HR | NA | 9.1 from recruitment  1.64 (1.21–2.23) |  |
| Keener 2018^25^ | USA | Inception | Population | 197/360 | Average 12.5 | Mortality risk | NA | 55% |  |
| Konno 2018^5^ | USA | Non-inception | Hospital | 350/980 | Range 0-22.9, median 3.7 | Mortality risk | NA | 36% | Median reported, but unclear exactly what it represents |
| Levy 2002^58^ | USA | Non-inception | Population | 41/180 | Mean 3.9 | Mortality risk | NA | 22.8% |  |
| Lo 2009^59^ | US | Inception | Population | 352/573 | 11 | Median, mortality risk | NA | Median 9y; 619% at 11 y |  |
| Montastruc 2001^60^ | France | Inception | Hospital (trial) | 17/58 | Mean 10.3 | Mortality risk; SMR | NA | 24% 10 y from start of treatment; 1.21 (0.71–1.95) | Excluded those aged >75 |
| Nakashima 1997^61^ | Japan | Non-inception | Population | 114/343 |  | Median | NA | 17 y in males, 22 y in females, from disease onset |  |
| Nam 2021^62^ | Korea | Inception | Population | NS/2657 | Up to 9.6 | KM survival | NA | 57% at 80 months |  |
| Nobrega 1967^63^ | USA | Inception | Population | 77/125 | Up to 32 | Median | NA | 12 y |  |
| Olanow 1998^64^ | USA and Europe | Inception | Hospital (trial) | 31/589 | Mean 4.1 | Mortality risk | NA | 5.37% at 4 y |  |
| Oosterveld 2015^65^ | Singpore | Inception (but retrospective) | Hospital | 363/1786 | Up to 31 | Mortality risk; Median (KM) survival | NA | 20.3%; 15.8 y | Some of the survival data is retrospective, hence bias towards those who have survived longer. |
| Ou 2020^66^ & Ou 2021^10^ | China | Inception | Hospital | 94/754 | Up to 14 | Mortality risk | NA | 47% at 10 y | KM in subset in Ou 2021; Almost no deaths first 5 years=Immortal time at start of study. Various co-morbidities excluded. |
| Peretz 2019^67^ | Israel | Inception | Population | NS/6622 | Mean 5.2 | Mortality risk | NA | 36% | Overlap with Chillag-Tamor cohort; data from KM plots |
| Pinter 2015^68^ | Austria | Inception | Hospital | 230/237 | Up to 40 | Mortality risk | NA | 97% at 38-40y | Partly retrospective. |
| Post 2011^69^ | Netherlands | Inception | Hospital | 6/126 | 3 | Mortality risk | NA | 4.8% at 3 y |  |
| Rajput 1984^70^ | USA | Inception | Population | NS/138 | NS | Median; RR vs controls | NA | 6y from dx; 1.6 at 5y, 1.6 at 10 y | Any degenerative parkinsonism included |
| Roos 1996^32^ | Netherlands | Inception | Hospital | 87/172 | Up to 12 | Median; RR | NA | 12y; RR 1.1 at 5 y 1.3 at 10 y |  |
| Santos-Garcia 2017^71^ | Spain | Non-inception | Hospital | 22/146 | 4 | Mortality risk | NA | 15.1% |  |
| Sato 2006^11^ | Japan | Inception | Hospital | NS/1397 | Up to 14 | Mortality risk | NA | 0.7% at 5 y; 10.2% at 10 y; 18.7% at 15 y |  |
| Sawada 2015^72^ | Japan | Non-inception | Hospital | 56/313 | Mean 4.8 | Mean survival | NA | 8.2 y | Mean only. Median not reached. |
| Schneider 2020^73^ | Brazil | Non-inception | Hospital | 62/150 | Average 8.9 | Mortality risk | NA | 41.3% | Denominator includes lost to FU (15%)–excluding them risk = 48% |
| Thaler 2018^74^ | Israel | Non-inception | Hospital | 380/1088 | NS | Median | NA | 20 y | From KM plot |
| Velseboer 2013^75^ | Netherlands | Inception | Hospital | 25/129 | 5 | Mortality risk | NA | 16.2% dead at 5 y |  |
| Wang 2015^76^ | China | Non-inception | Hospital | 11/157 | 5 | Mortality risk, SMR | NA | 7%; 0.62 (0.32–1.07) | Shorter FU of Zhang 2018 |
| Williams-Gray 2013^12^ | UK | Inception | Population | 63/142 | 10 | KM probability  Median time  SMR | NA | 45% at 10 y  10.3 y  1.29 (0.97–1.61) | CamPaIGN study, included in PICC. |
| Wills 2016^77^ | US | Non-inception | Hospital | 76/1673 | 6 | Mortality risk | NA | 5% |  |
| Wu 2016^34^ | Taiwan | Inception | Population | 293/1213 | Mean 6.3 | Incidence rate | NA | 38.3/1000py; 24% after mean 6.3 y | Population study |
| Xie 2021^78^ | USA | Non-inception | Population | 431/2033 | Up to 11 | KM probability | NS | 33% at 5y; 49% at 10y | From KM plot |
| Zhang 2018^79^ | China | Non-inception | Hospital | 31/157 | 10 | Mortality risk; SMR | NA | 19.7%; 0.87 (0.59–1.25) | Longer FU of Wang 2015 |

Abbreviations: ACE-R=Addenbrooke's Cognitive Examination-Revised; ADLs=activities of daily living; CDR=Clinical Dementia Rating; DSM=Diagnostic and Statistical Manual; dx=diagnosis; HR=hazards ratio; HY3 Hoehn & Yahr stage 3; ICD=International Classification of Diseases; FU=follow-up; KM=Kaplan-Meier; MDS=Movement Disorders Society; MMSE=minimental state examination; NA=not applicable; NH=nursing home; NS=not stated; PICC=Parkinson’s incidence cohorts collaboration; py=person years; RR=relative risk; S&E=Schwab and England; SMR=standardised mortality ratio; y=years.

**Supplemental Table 6: Results of systematic search** **of published literature: previous studies reporting prognostic factors for functional dependency, postural instability dementia, and mortality**

| **Study** | **Inception study?** | **Modelling method** | **Definition of outcome** | **Significant Prognostic factors (with odds ratios/hazard ratios)** | **Non-significant prognostic factors** | **Adjustment for confounders/other notes** |
| --- | --- | --- | --- | --- | --- | --- |
| **Prognostic factors for dependency** | | | | | | |
| Bjornestad 2016^2^ | Inception | Cox regression | Based on clinical interview | Age, 1.07 (1.03–1.11) per year  UPDRS Motor score, 1.04 (1.01–1.07) per unit  Motor symptom duration, HR 0.82 (0.69–0.98) per year  Presence of MCI, HR 1.81 (1.01–3.26) | Sex  Comorbidity score  Depressive symptoms | Multivariable model, adjusted for all factors in the model. NYPUM study, included in PICC. |
| Jasinka-Myga 2012^4^ | Non-inception | Cox regression | Based on clinical interview | Intellectual impairment, 3.55 (2.14–5.98)  Hoehn and Yahr stage (>2), 1.99 (1.42–2.80)  ADL score (any impairment), 2.13 (1.47–4.11)  UPDRS rigidity (5-point increase), 1.26 (1.02–1.55)  UPDRS bradykinesia (10-point increase), 1.38 (1.13–1.70)  UPDRS instability (3-point increase), 1.73 (1.43–2.09)  UPDRS dyskinesia (>0), 2.39 (1.61–3.57)  UPDRS total (20-point increase), 1.44 (1.14–1.82) | Gender, personal history of depression, current depression, family history of movement disorders, family history of PD, UPDRS—tremor (5-point increase) | Adjusted for age and disease duration |
| Kim 2020^80^ | Inception | Cox regression | Not stated | Obesity (P=0.001) | Overweight | Multivariable model, other factors not stated in abstract. Full text unavailable. |
| Macleod 2016^6^ | Inception | Cox regression | S&E <80% | Age at diagnosis (10-year increase), 2.57 (1.95–3.39)  Smoking history (10-pack-year increase), 1.18 (1.08–1.28)  Charlson score (1-point increase), 1.24 (1.08–1.43)  UPDRS bradykinesia score (5-point increase), 1.54 (1.24–1.90)  UPDRS Axial score (5-point increase), 1.28 (1.69–3.06)  UPDRS motor score (10-unit increase), 1.55 (1.28–1.87)  MMSE score (1-point increase), 0.78 (0.70–0.87)  Hoehn & Yahr stage , 1.80 (1.33–2.42) | Sex  Deprivation score | Multivariable model, adjusted for all factors in the model. PINE study, included in PICC. |
| Santos Garcia 2021^7^ | Non-inception | Univariable tests | S&E <80% | Male sex, disease duration, number of antipark drugs, LEDD, UPDRS part III, UPDRS par IV, FOGQ, non-tremor phenotype, PD-CRS, NMSS, BDI-II, PDSS, NPI, VAS-PAIN, VASF-physical, VASF-mental, PDQ39, PQ10, EUROHIS-QOL8, Schwab & England | Age at baseline  QUIP-RS | Univariable only (multivariable predictors of worsening S&E reported in paper) |
| **Prognostic factors for postural instability** | | | | | | |
| Hiorth 2017^9^5 | Inception | Generalized estimating equations | Any falls | Age,OR per year, 1.04 (1.01–1.07)  UPDRS motor score, OR 1.03 (1.01–1.04)  PIGD phenotype, OR 4.32 (3.21–5.8)  PD dementia, OR 2.03 (1.05–3.94) | Sex, disease duration, freezing of gait, BMI, MADRS score, MMSE score, LED. | Adjusted for significant factors and disease duration. ParkWest study, included in PICC. |
| Williams-Gray 2013^12^ | Inception | Cox regression | HY3 | Age, HR per year=1.05  Tremor-dominant phenotype, HR=4.07  Greater comorbidity, HR=1.33, | Sex, smoking status, UPDRS motor score, motor phenotype, depression, equivalent levodopa dose, verbal IQ, various neuropsychological variables, MAPT H1/H1, SNCA, APOE ε4 carrier, COMT gene | Adjusted for other significant variables. CamPaIGN study, included in PICC. |
| **Prognostic factors for dementia** | | | | | | |
| Anang 2014^13^ | Non-inception | Logistic regression | Level 2 MDS criteria | Age, 1.14 (1.07–1.21)  Male sex, 1.04 (0.94-1.14)  Any falls, 6.87 (2.22–21.3)  Freezing, 5.81 (1.87–18.0)  Gait impairment, 1.15 (1.06–1.26)  RBD, 49.7 (7.4–333.5)  MCI, 18.13 (3.96–83.05)  Hallucinations, 10.20 (2.40–44.00)  Orthostatic symptoms, 3.16 (1.60–6.51) |  | Adjusted for baseline age, sex, baseline disease duration, and duration of follow-up). |
| Anang 2017^14^ | Non-inception | Logistic regression | MDS PDD criteria | Age≥70 5.2 (1.9–14.6)  Male sex, 4.1 (1.6–10.5)  RBD, 5.4 (1.9–14.9)  SBP drop, 5.3 (1.5–19.5),  MCI, 9.2 (1.8–46.0) |  | Adjusted for baseline age, sex, disease duration, and duration of follow-up |
| Åström 2022^15^ | Inception | Cox regression | ICD-10 coding | HY >2 vs <2, 1.6 (1.1–2.3) |  | Adjusted for: sex, year of birth, family history, socio-economic status, marital status |
| Bäckström 2022^16^ | Inception | Cox regression | MDS PDD criteria | Age, 0.05 (1.01–1.09)  UPDRS III subscore, 1.0 (1.0–1.1)  MCI, 4.2 (2.2–7.7)  Olfactory function, 0.8 (0.7–0.9)  CSF NfL to Aβ42 ratio, 1.5 (1.2–1.7)  DAT uptake, most affected caudate, 0.59 (0.39–0.90) | Years of education, smoker, MADRS score, sex, H&Y stage, UPDRS total score, PIGD score, tremor score, blink frequency, slow saccades, postural instability first year, symmetrical onset, MCI, alcohol use, orthostatic blood pressure drop, BMI, APOE ɛ4 genotype, MAPT haplotype, CSF NfL, CSF Aβ42, CSF α-synuclein, TSH, leucocytes, B12 vitamin, folate, DAT uptake in putamen. | Adjusted for other significant variables in the model. NYPUM study, included in PICC. |
| Counsell 2022^21^ | Inception | Cox regression | DSM-IV | Age (10-year increase), 2.36 (1.72–3.25)  History of cognitive symptoms, 1.98 (1.16–3.40)  History of dream enactment symptoms, 2.63 (1.54–4.49)  MMSE, 0.89 (0.81–0.98) | Sex, years of education, smoking status, BMI, history of vascular disease or diabetes, history of cognitive symptoms, history of dream enactment symptoms, MMSE, UPDRS part 3 (motor) | Adjustment for all variables in the model. PINE study, included in PICC. |
| Hughes 2000^23^ | Non-inception | Cox regression | DSM-III-R | Age, 1.07 (0.99–1.15)  Webster score, 1.16 (1.03–1.31) | PD duration, age at onset, sex, NUDS, MADRS, WAIS, NART | Adjusted for age and Webster score |
| Janvin 2005^24^ | Non-inception | Logistic regression | DSM-III-R | Time on the SWT–third card, 1.03 (1.01–1.04) | Education, sex, age, age at onset, disease duration, Hoehn & Yahr stage |  |
| Keener 2018^25^ | Inception | Cox regression | MMSE <24 | Postural Reflex Impairment Score, 1.38 (1.16, 1.64) | Age at diagnosis, sex, non-European ancestry, education, pack-years, PD Duration, UPDRS III, resting tremor score, bradykinesia score, rigidity score, PD subtype, levodopa use/LED, MMSE, GDS, diabetes, stroke, heart attack, high BP, systolic BP, diastolic BP, BMI, sleep Levels | Adjusted for age at diagnosis, sex, European ancestry, education, and smoking |
| Levy 2000^81^ | Non-inception | Cox regression |  | Male sex, 2.57 (1.43–4.61)  Age at baseline, 1.09 (1.05–1.13)  Education, 0.93 (0.88–0.99)  UPDRS Levy B, 1.19 (1.09–1.30)  Bradykinesia, 1.09 (1.01–1.18) | Duration of PD, use of dopamine agonists, use of anticholinergics, UPDRS subscore A | Same cohort as Levy 2002  Adjusted for all variables in model |
| Levy 2002^58^ | Non-inception | Cox regression | DSM-III-R | Some smoking variables | Head injury, hypertension, diabetes mellitus, alcohol, coffee | Same cohort as Levy 2000  Adjusted for age at baseline, sex, years of education, PD duration, UPDRS part III |
| Stern 1993^33^ | Non-inception | Cox regression | DSM III | Masked facies, depression, confusion or psychosis on levodopa, age>69,  Higher CUPDRS scores (>24) | Various | HR not reported |
| Williams-Gray 2013^12^ | Inception | Cox regression | DSM-IV | Age, HR (per year)=1.08  Semantic fluency <20, HR=3.05  Impaired pentagon copying, HR=2.55  UPDRS motor score ≥25, HR=3.18  MAPT H1/H1, HR=3.08 | Sex, smoking status, motor phenotype, depression, equivalent levodopa dose, verbal IQ, other neuropsychological variables, SNCA gene, APOE ε4 carrier, COMT gene | Adjusted for other significant variables in the model, except for MAPT genotype (only adjusted for age) |
| Wu 2016^34^ | Inception | Cox regression | ICD-9 coding | Age at diagnosis, 1.08 (1.06–1.09)  Diabetes, 1.58, (1.08–2.29)  Constipation before PD diagnosis, 1.51 (1.08–2.12) | Sex, RBD, depression | Adjusted for age, gender, hypertension, diabetes mellitus, hyperlipidemia and ischemic heart disease |
| **Prognostic factors for death** | | | | | | |
| Bäckström 2018^35^ | Inception | Cox regression | NA | Age, 1.11 (1.07-1.16)  PIGD score, 3.25 (1.75–6.05)  Severity of hyposmia, 0.84 (0.75–0.93)  MCI, 1.81 (1.04–3.14)  Inflammatory CSF reaction, 5.59 (2.67–11.71) | Disease duration, sex, UPDRS total score, UPDRS part III score, tremor score, freezing, falling, dysphagia, BMI, timed-up-and-go test, previous smoker, MADRS depression score, years of education, dementia within first 3 years, orthostatic BP drop, APOE ε4 genotype, CSF biomarkers, CSF leukocytes, DAT uptake in most affected putamen. | Adjusted for other significant variables in model. NYPUM study, included in PICC. |
| Bugalho 2019^39^ | Non-inception | Cox regression | NA | S&E, 0.98 (0.95–1.00) | LEDD, UPDRS II, UPDRS III, Hoehn & Yahr, motor subtype, MCI, dementia, MoCA, NMSS, RBDSQ ≥ 6, EQ-index, EQ-VAS, CCI | Adjusted for age, gender and disease duration |
| Chillag-Talmor 2013^41^ | Inception | Cox regression | NA | Age, 1.089 (1.080–1.098)  Male sex, 1.40 (1.25–1.56) |  | Adjusted for age/sex |
| De Lau 2014^44^ | Non-inception | Cox regression | NA | Male sex, 3.2 (1.4–7.0)  Age at recruitment (per year increase), 1.1 (1.1–1.2)  Cognitive impairment (presence vs. absence), 3.7 (1.9–7.2)  PIGD score (per unit increase), 1.3 (1.1–1.5)  Psychotic symptoms (presence vs. absence), 3.7 (1.4–9.6) | Disease duration, age at onset, H&Y stage, LED, tremor, bradykinesia/rigidity, presence of freezing, depression, autonomic dysfunction, excessive daytime sleepiness, motor fluctuations, dyskinesias | Adjusted for other significant variables in the model |
| Duarte 2013^47^ | Inception | Cox regression | NA | Age, 1.05 (1.01 –1.09)  Initial presentation of akinesia + rigidity, 2.20 (1.06–4.88) | Dopamine agonist treatment | Adjusted for all other variables in model |
| Gonzalez 2022^52^ | Inception | Cox regression | NA | Age, 2.20 (1.52–3.17)  UPDRS motor score, 1.02 (1.01–1.05)  PIGD, 1.83 (1.09–3.01) | Sex, smoking, baseline HY stage, MMSE score, MADRS score, CCI | Adjusted for other significant variables in the model. ParkWest study, included in PICC. |
| Gray 2009^82^ | Non-inception | Cox regression | NA | Male gender 2.86 (1.52–5.41))  Age at recruitment 1.07 (1.02–1.12)  Tinetti gait score 1.30 (1.14–1.49) |  | Adjusted for each variable in model. |
| Hoogland 2019^56^ | Inception | Cox regression | NA | Age (per year), 1.11 (1.07–1.15)  LED (per 100 mg), 1.20 (1.01–1.42)  PD-MCI, 1.76 (1.04–2.97) | UPDRS-III A (per point), 0.98 (0.94–1.03)  UPDRS-III B (per point), 1.11 (0.96–1.27)  MMSE (per point), 1.11 (0.96–1.28) | Adjusted for all other variables in model |
| Keener 2018^25^ | Inception | Cox regression | NA | Age at diagnosis, 1.09 (1.07–1.11)  Education, 0.91 (0.85–0.97)  Pack-years, 1.01 (1.00–1.01)  UPDRS III, 1.07 (1.05–1.10)  Resting tremor score, 1.13 (1.04–1.22)  Bradykinesia score, 1.20 (1.12–1.28)  Rigidity score, 1.14 (1.07–1.20)  Postural instability score, 1.29 (1.18–1.41)  MMSE, 0.79 (0.72–0.87)  GDS, 1.18 (1.08–1.29)  Heart Attack, 1.87 (1.23–2.84) | Sex, non-European ancestry, PD duration, PD subtype, levodopa use/LED, diabetes, stroke, high BP, systolic BP, diastolic BP, BMI, average sleep levels | Adjusted for age at diagnosis, sex, European ancestry, education, and smoking |
| Marras 2005^83^ | Non-inception | Cox regression | NA | Age, 10 yr increase (p<0.001)  H&Y stage (p=0.003)  Male gender 1.36 (1.02–1.81)  Smoking hx, 10-pack yr increase 1.09 (1.03–1.14)  Full-time employment 0.59 (0.41–0.86)  Cardiac co-morbidity 1.44 (1.10–1.91)  Extensor of equivocal plantar response on either side 1.45 (p=0.02)  Total UPDRS, 10 point increase 1.17 (1.01–1.36) | Pulmonary comorbidity, urologic comorbidity, MMSE, symmetry score, postural instability gait disorder score, duration of symptoms, tremor as initial symptom | Adjusted for other significant factors in the model. |
| Nam 2021^62^ | Inception | Cox regression | NA | Low-middle income quartile vs lowest income quartile, 0.72 (0.49–1.04) | Other income quartiles  Rural vs urban living | NS |
| Oosterveld 2015^65^ | Inception | Cox regression | NA | Age at diagnosis. 1.06 (1.03–1.08)  Male sex, 2.30 (1.57–3.35)  MMSE <24, 2.30 (1.55–3.41)  UPDRS motor score>29, 1.63 (1.13–2.35)  Bradykinesia subscore, 1.05 (1.01–1.09) | Ethnicity, education level, tremor subscore, rigidity subscore, PIGD subscore, use of levodopa, use of amantadine, use of trihexyphenidyl, use of agonist, use of selegiline. | Adjusted for age at diagnosis, gender, ethnicity, years of education, MMSE, UPDRS motor score and the use of different types of medication |
| Peretz 2019^67^ | Inception | Cox regression | NA | Age  Male sex, 1.39 (1.28–1.52) |  | Adjusted for age and sex |
| Pinter 2015^68^ | Inception | Cox regression | NA | Age at onset, 1.08 (1.07, 1.10)  Male sex, 1.55 (1.19, 2.02)  Tremor at onset, 0.51 (0.37, 0.70)  Asymmetry at onset, 0.56 (0.38, 0.84)  Gait disorder at onset, 1.61 (1.11, 2.36) |  | Adjusted for age |
| Sawada 2015^72^ | Non-inception | Cox regression | NA | Adjusted HR per two-fold higher CRP concentration 1.29 (1.10-1.52), |  | Adjusted for age, sex, PD duration, modified HohenH&Y stages, MMSE scores, and serum albumin |
| Schneider Medeiros 2020^73^ | Non-inception | Cox regression | NA | Occupational pesticide exposure, 2.23 (1.09–4.59) | Ever smoker, disease duration at recruitment, female sex, caffeine | Adjusted for all variables in model. Age scale for survival analysis. |
| Wang 2015^76^ | Non-inception | Logistic regression | NA | MMSE, 0.78 (0.63–0.97)  Epworth sleep scale, 1.24 (1.01–1.51) | Age at onset, independent living,PD sleep scale | Adjusted for age and sex |
| Weintraub 2016^84^ | Non-inception | Cox regression | NA | Antipsychotic use, 2.35 (2.08–2.66) |  | Adjusted for age, sex, race, dementia, time from PD diagnosis, previous delirium, previous hospitalization, CCI, |
| Williams-Gray 2013^12^ | Inception | Cox regression | NA | Age at diagnosis, HR=1.08  Smoking history, HR=2.07 | Sex, UPDRS motor score, motor phenotype, co-morbidity, depression, equivalent levodopa dose, verbal IQ, various neuropsychological variables, MAPT H1/H1, SNCA, APOE ε4 carrier, COMT gene | Adjusted for other significant variables in the model. |
| Wills 2016^77^ | Non-inception | Cox regression | NA | NS | BMI | Adjusted for age, sex, disease duration, total UPDRS score, history of cardiovascular disease |
| Wu 2016^34^ | Inception | Cox regression | NA | Age at diagnosis, 1.07 (1.06–1.08)  Male sex, 1.49 (1.17–1.91)  Diabetes, 1.49 (1.12–1.97)  Chronic kidney disease (HR not reported)  Constipation, 1.45 (1.12–1.87)  RBD, 2.71 (1.10–6.65 | Depression | Adjusted for age, gender, hypertension, diabetes mellitus, hyperlipidaemia and ischemic heart disease |
| Xie 2021^78^ | Non-inception | Cox regression | NA | Black race, 1.6 (1.2–2.1)  Age >71.9, 2.4 (1.8–3.2)  Inpatient admission, 2.6 (1.3–5.5)  Malignancy, 1.4 (1.0–1.9)  Amantadine use, 0.4 (0.2–0.8)  MAOB-I use, 0.2 (0.1–0.5)  Dopamine agonist use, 0.5 (0.3–0.8)  Antipsychotic use, 0.6 (0.5–0.9) |  | Adjusted for other variables in model |
| Zhang 2022^85^ | Inception | Cox regression | NA | Highest vs the lowest diet quality quartile, 0.69 (95%CI, 0.56- 0.85)  Physical activity, highest versus lowest quartile, 0.71(0.57-0.87) |  | Age, smoking status, BMI, total energy intake, caffeine intake, NSAID use, hypertension, type 2 diabetes, hypercholesterolemia, and postmenopausal hormone use in women |

Abbreviations: APOE=apolipoprotein E; BMI=body mass index; CCI=Charlson comorbidity index; COMT=catechol-O-methyl transferase; DaT=dopamine transporter; DSM=Diagnostic and Statistical Manual; GDS=geriatric depression scale; HR=hazards ratio; HY=Hoehn & Yahr; HY3 Hoehn & Yahr stage 3; ICD=International Classification of Diseases; LED=levodopa-equivalent dose; MADRS=Montgomery-Asberg depression rating scale; MAPT=microtubule-associated protein tau; MDS=Movement Disorders Society; MMSE=minimental state examination; NA=not applicable; NS=not stated; NSAID=non-steroidal anti-inflammatory drug; PD=Parkinson’s disease; PICC=Parkinson’s incidence cohorts collaboration; PIGD=postural instability and gait difficulties; QUIP=questionnaire for impulsive-compulsive disorders in Parkinson’s disease; RBD=REM sleep behaviour disorder; SNCA=alpha synuclein gene; y=years.

**Supplemental Table 7: Previous PICC publications on dependency, postural instability, dementia, and mortality**

| **Author & year** | **Outcomes reported** | **Analyses** | **PICC studies** |
| --- | --- | --- | --- |
| Bäckström 2015^86^ | Dementia | Risk, predictors | NYPUM |
| Bäckström 2018^35^ | Mortality | Risk, predictors | NYPUM |
| Bäckström 2022^16^ | Dementia | Risk, predictors | NYPUM |
| Bjornestad 2016^2^ | Dependency | Risk, predictors | ParkWest |
| Bjornestad 2017^38^ | Dementia, mortality | Risk, predictors | ParkWest |
| Borda 2022^87^ | Dementia | Association between frailty and dementia | ParkWest |
| Camacho 2021^88^ | Dementia | Association between constipation and dementia | ParkWest, PICNICS |
| Counsell 2022^21^ | Dementia | Risk, predictors | PINE |
| Fielding 2016^50^ | Dependency, mortality | Risk, predictors | PINE |
| Gonzalez 2022^52^ | Mortality | Risk, predictors | ParkWest |
| Hiorth 2017^9^ | Postural instability | Risk, predictors | ParkWest |
| Hiorth 2024^89^ | Dementia | Association between orthostatic hypotension and dementia | ParkWest |
| Lunde 2018^90^ | Dementia | Association between GBA and dementia | NYPUM, ParkWest, PINE |
| Macleod 2016^6^ | Dependency | Risk, predictors | PINE |
| Oftedal 2023^91^ | Dementia | Association between CSF Glucocerebrosidase activity and dementia | ParkWest |
| Pedersen 2013^92^ | Dementia | Association between MCI and dementia | ParkWest |
| Pedersen 2017^93^ | Dementia | Association between MCI and dementia | ParkWest |
| Stoker 2020^94^ | Dementia, postural instability, mortality | Risk, GBA1 variants as predictors | CamPaIGN, PICNICS |
| Szwedo 2022^95^ | Dementia | APOE and GBA variants as predictors | All PICC studies |
| Williams-Gray 2009^96^ | Dementia | APOE as predictor | CamPaIGN |
| Williams-Gray 2009^97^ | Dementia | Risk, clinical and genetic predictors | CamPaIGN |
| Williams-Gray 2013^12^ | Mortality, dementia, postural instability | Risk, clinical and genetic predictors | CamPaIGN |
| Winder-Rhodes 2013^98^ | Dementia, postural instability | GBA variants as predictors | CamPaIGN, PICNICS |

**Supplemental Table 8: Association between sex and mortality in inception studies in Parkinson’s disease**

| **Study/year** | **Measure of association,** | **Association (hazard ratio [95% confidence interval] unless stated otherwise)** |
| --- | --- | --- |
| Chillag-Talmor 2013^1^ | Hazard ratio, male vs female | 1.40 (1.25–1.56) |
| Hely 2008^2^ | Not stated | Non-significant higher mortality in men |
| Keener 2018^3^ | Hazard ratio, male vs female | 1.01 (0.76–1.36) |
| Lo 2009^4^ | Hazard ratio, male vs female | 1.19 (0.96–1.48) |
| Oosterveld 2015^5^ | Hazard ratio, male vs female | 2.29 (1.57–3.35) |
| Peretz 2019^6^ | Hazard ratio, male vs female | 1.39 (1.28–1.52) |
| Pinter 2015^7^ | Hazard ratio, male vs female | 1.55 (1.19–2.02) |
| Roos 1996^8^ | Not stated | Only p-value of 0.1 |
| Sato 2006^9^ | P-value only from logistic regression | Higher mortality in men, p=0.025 |
| Willis 2012^10^ | Hazard ratio, male vs female | 1.35 (1.33–1.37) |
| Wu 2016^11^ | Hazard ratio, male vs female | 1.49 (1.17–1.91) |
| Current study | Hazard ratio, male vs female | 1.37 [1.11–1.69) |

**Supplemental References**

1. Auyeung M, Tsoi TH, Mok V, et al. Ten year survival and outcomes in a prospective cohort of new onset Chinese Parkinson's disease patients. *Journal of neurology, neurosurgery, and psychiatry* 2012; **83**: 607-11.

2. Bjornestad A, Tysnes O-B, Larsen JP, Alves G. Loss of independence in early Parkinson disease: A 5-year population-based incident cohort study. *Neurology* 2016; **87**: 1599-606.

3. Hely MA, Reid WGJ, Adena MA, Halliday GM, Morris JGL. The Sydney multicenter study of Parkinson's disease: the inevitability of dementia at 20 years. *Movement disorders : official journal of the Movement Disorder Society* 2008; **23**: 837-44.

4. Jasinska-Myga B, Heckman MG, Wider C, Putzke JD, Wszolek ZK, Uitti RJ. Loss of ability to work and ability to live independently in Parkinson's disease. *Parkinsonism & related disorders* 2012; **18**: 130-5.

5. Konno T, Deutschlander A, Heckman MG, et al. Comparison of clinical features among Parkinson's disease subtypes: A large retrospective study in a single center. *Journal of the neurological sciences* 2018; **386**: 39-45.

6. Macleod AD, Counsell CE. Predictors of functional dependency in Parkinson's disease. *Movement Disorders* 2016; **31**: 1482-8.

7. Santos García D, de Deus Fonticoba T, Cores Bartolomé C, et al. Predictors of Loss of Functional Independence in Parkinson’s Disease: Results from the COPPADIS Cohort at 2-Year Follow-Up and Comparison with a Control Group. *Diagnostics* 2021; **11**: 1801.

8. Scigliano G, Musicco M, Soliveri P, et al. Mortality associated with early and late levodopa therapy initiation in Parkinson's disease. *Neurology* 1990; **40**: 265-9.

9. Hiorth YH, Alves G, Larsen JP, Schulz J, Tysnes O-B, Pedersen KF. Long-term risk of falls in an incident Parkinson's disease cohort: the Norwegian ParkWest study. *Journal of neurology* 2017; **264**: 364-72.

10. Ou R, Wei Q, Hou Y, et al. Effect of diabetes control status on the progression of Parkinson's disease: A prospective study. *Annals of clinical and translational neurology* 2021; **8**: 887-97.

11. Sato K, Hatano T, Yamashiro K, et al. Prognosis of Parkinson's disease: time to stage III, IV, V, and to motor fluctuations. *Movement Disorders* 2006; **21**: 1384-95.

12. Williams-Gray CH, Mason SL, Evans JR, et al. The CamPaIGN study of Parkinson's disease: 10-year outlook in an incident population-based cohort. *Journal of neurology, neurosurgery, and psychiatry* 2013; **84**: 1258-64.

13. Anang JBM, Gagnon J-F, Bertrand J-A, et al. Predictors of dementia in Parkinson disease: a prospective cohort study. *Neurology* 2014; **83**: 1253-60.

14. Anang JBM, Nomura T, Romenets SR, Nakashima K, Gagnon J-F, Postuma RB. Dementia Predictors in Parkinson Disease: A Validation Study. *Journal of Parkinson's disease* 2017; **7**: 159-62.

15. Astrom DO, Simonsen J, Raket LL, et al. High risk of developing dementia in Parkinson's disease: a Swedish registry-based study. *Scientific reports* 2022; **12**: 16759.

16. Bäckström D, Granåsen G, Mo SJ, et al. Prediction and early biomarkers of cognitive decline in Parkinson disease and atypical parkinsonism: a population-based study. *Brain communications* 2022; **4**.

17. Bakeberg MC, Gorecki AM, Kenna JE, et al. Differential effects of sex on longitudinal patterns of cognitive decline in Parkinson's disease. *Journal of neurology* 2021; **268**: 1903-12.

18. Biggins CA, Boyd JL, Harrop FM, et al. A controlled, longitudinal study of dementia in Parkinson's disease. *Journal of neurology, neurosurgery, and psychiatry* 1992; **55**: 566-71.

19. Buter TC, van den Hout A, Matthews FE, Larsen JP, Brayne C, Aarsland D. Dementia and survival in Parkinson disease: a 12-year population study. *Neurology* 2008; **70**: 1017-22.

20. Campbell MC, Myers PS, Weigand AJ, et al. Parkinson disease clinical subtypes: key features & clinical milestones. *Annals of clinical and translational neurology* 2020; **7**: 1272-83.

21. Counsell C, Giuntoli C, Khan QI, Maple-Grødem J, Macleod AD. The incidence, baseline predictors, and outcomes of dementia in an incident cohort of Parkinson's disease and controls. *Journal of neurology* 2022; **269**: 4288-98.

22. de Lau LML, Schipper CMA, Hofman A, Koudstaal PJ, Breteler MMB. Prognosis of Parkinson disease: risk of dementia and mortality: the Rotterdam Study. *Archives of neurology* 2005; **62**: 1265-9.

23. Hughes TA, Ross HF, Musa S, et al. A 10-year study of the incidence of and factors predicting dementia in Parkinson's disease. *Neurology* 2000; **54**: 1596-602.

24. Janvin CC, Aarsland D, Larsen JP. Cognitive predictors of dementia in Parkinson's disease: a community-based, 4-year longitudinal study. *Journal of geriatric psychiatry and neurology* 2005; **18**: 149-54.

25. Keener AM, Paul KC, Folle A, Bronstein JM, Ritz B. Cognitive Impairment and Mortality in a Population-Based Parkinson's Disease Cohort. *Journal of Parkinson's disease* 2018; **8**: 353-62.

26. Kwon K-Y, Kang SH, Kim M, et al. Nonmotor Symptoms and Cognitive Decline in de novo Parkinson's Disease. *The Canadian journal of neurological sciences Le journal canadien des sciences neurologiques* 2014; **41**: 597-602.

27. Levy G, Tang M-X, Cote LJ, et al. Do risk factors for Alzheimer's disease predict dementia in Parkinson's disease? An exploratory study. *Movement disorders : official journal of the Movement Disorder Society* 2002; **17**: 250-7.

28. Liu C-C, Sun Y, Lee P-C, Li C-Y, Hu SC. Risk of dementia after Parkinson's disease in Taiwan: a population-based retrospective cohort study using National Health Insurance claims. *BMJ open* 2019; **9**: e025274.

29. Malek N, Lawton MA, Swallow DMA, et al. Vascular disease and vascular risk factors in relation to motor features and cognition in early Parkinson's disease. *Movement disorders : official journal of the Movement Disorder Society* 2016; **31**: 1518-26.

30. Nicoletti A, Luca A, Baschi R, et al. Vascular risk factors, white matter lesions and cognitive impairment in Parkinson's disease: the PACOS longitudinal study. *Journal of neurology* 2021; **268**: 549-58.

31. Real R, Martinez-Carrasco A, Reynolds RH, et al. Association between the LRP1B and APOE loci and the development of Parkinson's disease dementia. *Brain* 2023; **146**: 1873-87.

32. Roos RA, Jongen JC, van der Velde EA. Clinical course of patients with idiopathic Parkinson's disease. *Movement disorders : official journal of the Movement Disorder Society* 1996; **11**: 236-42.

33. Stern Y, Marder K, Tang MX, Mayeux R. Antecedent clinical features associated with dementia in Parkinson's disease. *Neurology* 1993; **43**: 1690-2.

34. Wu Y-H, Lee W-J, Chen Y-H, Chang M-H, Lin C-H. Premotor Symptoms as Predictors of Outcome in Parkinsons Disease: A Case-Control Study. *PloS one* 2016; **11**: e0161271.

35. Bäckström D, Granåsen G, Domellöf ME, et al. Early predictors of mortality in parkinsonism and Parkinson disease: A population-based study. *Neurology* 2018; **91**: e2045-e56.

36. Ben-Shlomo Y, Marmot MG. Survival and cause of death in a cohort of patients with parkinsonism: possible clues to aetiology? *Journal of neurology, neurosurgery, and psychiatry* 1995; **58**: 293-9.

37. Berger K, Breteler MM, Helmer C, et al. Prognosis with Parkinson's disease in europe: A collaborative study of population-based cohorts. Neurologic Diseases in the Elderly Research Group. *Neurology* 2000; **54**: S24-7.

38. Bjornestad A, Pedersen KF, Tysnes O-B, Alves G. Clinical milestones in Parkinson's disease: A 7-year population-based incident cohort study. *Parkinsonism & related disorders* 2017; **42**: 28-33.

39. Bugalho P, Ladeira F, Barbosa R, et al. Motor and non-motor function predictors of mortality in Parkinson's disease. *Journal of neural transmission (Vienna, Austria : 1996)* 2019; **126**: 1409-15.

40. Chia LG, Liu LH. Parkinson's disease in Taiwan: an analysis of 215 patients. *Neuroepidemiology* 1992; **11**: 113-20.

41. Chillag-Talmor O, Giladi N, Linn S, et al. Estimation of Parkinson's disease survival in Israeli men and women, using health maintenance organization pharmacy data in a unique approach. *Journal of neurology* 2013; **260**: 62-70.

42. D'Amelio M, Ragonese P, Morgante L, et al. Long-term survival of Parkinson's disease: a population-based study. *Journal of neurology* 2006; **253**: 33-7.

43. Das SK, Misra AK, Ray BK, et al. Epidemiology of Parkinson disease in the city of Kolkata, India: a community-based study. *Neurology* 2010; **75**: 1362-9.

44. de Lau LML, Verbaan D, Marinus J, van Hilten JJ. Survival in Parkinson's disease. Relation with motor and non-motor features. *Parkinsonism & related disorders* 2014; **20**: 613-6.

45. Diamond SG, Markham CH, Hoehn MM, McDowell FH, Muenter MD. Multi-center study of Parkinson mortality with early versus later dopa treatment. *Annals of neurology* 1987; **22**: 8-12.

46. Driver JA, Kurth T, Buring JE, Gaziano JM, Logroscino G. Parkinson disease and risk of mortality: a prospective comorbidity-matched cohort study. *Neurology* 2008; **70**: 1423-30.

47. Duarte J, Garcia Olmos LM, Mendoza A, Claveria LE. The natural history of Parkinson's disease in the province of Segovia: mortality in a longitudinal study (20-year follow-up). *Acta neurologica Scandinavica* 2013; **127**: 295-300.

48. Elbaz A, Bower JH, Peterson BJ, et al. Survival study of Parkinson disease in Olmsted County, Minnesota. *Archives of neurology* 2003; **60**: 91-6.

49. Fall P-A, Saleh A, Fredrickson M, Olsson J-E, Granerus A-K. Survival time, mortality, and cause of death in elderly patients with Parkinson's disease: a 9-year follow-up. *Movement disorders : official journal of the Movement Disorder Society* 2003; **18**: 1312-6.

50. Fielding S, Macleod AD, Counsell CE. Medium-term prognosis of an incident cohort of parkinsonian patients compared to controls. *Parkinsonism & related disorders* 2016; **32**: 36-41.

51. Forsaa EB, Larsen JP, Wentzel-Larsen T, Alves G. What predicts mortality in Parkinson disease?: a prospective population-based long-term study. *Neurology* 2010; **75**: 1270-6.

52. Gonzalez MC, Dalen I, Maple-Grodem J, Tysnes O-B, Alves G. Parkinson's disease clinical milestones and mortality. *NPJ Parkinson's disease* 2022; **8**: 58.

53. Guttman M, Slaughter PM, Theriault ME, DeBoer DP, Naylor CD. Parkinsonism in Ontario - Increased mortality compared with controls in a large cohort study. *Neurology* 2001; **57**: 2278-82.

54. Hobson P, Meara J. Mortality and quality of death certification in a cohort of patients with Parkinson's disease and matched controls in North Wales, UK at 18 years: a community-based cohort study. *BMJ open* 2018; **8**: e018969.

55. Hoehn MM, Yahr MD. Parkinsonism: onset, progression and mortality. *Neurology* 1967; **17**: 427-42.

56. Hoogland J, Post B, de Bie RMA. Overall and Disease Related Mortality in Parkinson's Disease - a Longitudinal Cohort Study. *Journal of Parkinson's disease* 2019; **9**: 767-74.

57. Hughes TA, Ross HF, Mindham RHS, Spokes EGS. Mortality in Parkinson's disease and its association with dementia and depression. *Acta neurologica Scandinavica* 2004; **110**: 118-23.

58. Levy G, Tang MX, Louis ED, et al. The association of incident dementia with mortality in PD. *Neurology* 2002; **59**: 1708-13.

59. Lo RY, Tanner CM, Albers KB, et al. Clinical features in early Parkinson disease and survival. *Archives of neurology* 2009; **66**: 1353-8.

60. Montastruc JL, Desboeuf K, Lapeyre-Mestre M, Senard JM, Rascol O, Brefel-Courbon C. Long-term mortality results of the randomized controlled study comparing bromocriptine to which levodopa was later added with levodopa alone in previously untreated patients with Parkinson's disease. *Movement disorders : official journal of the Movement Disorder Society* 2001; **16**: 511-4.

61. Nakashima K, Maeda M, Tabata M, Adachi Y, Kusumi M, Ohshiro H. Prognosis of Parkinson's disease in Japan. Tottori University Parkinson's Disease Epidemiology (TUPDE) Study Group. *European neurology* 1997; **38 Suppl 2**: 60-3.

62. Nam JS, Kim YW, Shin J, Chang JS, Yoon SY. Hip Fracture in Patients with Parkinson's Disease and Related Mortality: A Population-Based Study in Korea. *Gerontology* 2021; **67**: 544-53.

63. Nobrega F, Glattre E, Kurland LT, Okazaki H. Comments on the epidemiology of parkinsonism including prevalence and incidence statistics for Rochester, Minnesota, 1935-1966. In: Barbeau A, Brunette JR, eds. Progress in Neurogenetics. Amsterdam: Excerpta Medica; 1967: 474-85.

64. Olanow CW, Myllyla VV, Sotaniemi KA, et al. Effect of selegiline on mortality in patients with Parkinson's disease: a meta-analysis. *Neurology* 1998; **51**: 825-30.

65. Oosterveld LP, Allen JC, Jr., Reinoso G, et al. Prognostic factors for early mortality in Parkinson's disease. *Parkinsonism & related disorders* 2015; **21**: 226-30.

66. Ou R, Wei Q, Hou Y, et al. Association between positive history of essential tremor and disease progression in patients with Parkinson's disease. *Scientific reports* 2020; **10**: 21749.

67. Peretz C, Rozani V, Giladi N, et al. The Modifying Effect of Age on Survival in Parkinson's Disease: A Population-Based Cohort Study. *Neuroepidemiology* 2019; **53**: 13-9.

68. Pinter B, Diem-Zangerl A, Wenning GK, et al. Mortality in Parkinson's disease: a 38-year follow-up study. *Movement disorders : official journal of the Movement Disorder Society* 2015; **30**: 266-9.

69. Post B, Muslimovic D, van Geloven N, et al. Progression and prognostic factors of motor impairment, disability and quality of life in newly diagnosed Parkinson's disease. *Movement Disorders* 2011; **26**: 449-56.

70. Rajput AH. Epidemiology of Parkinson's disease. *The Canadian journal of neurological sciences Le journal canadien des sciences neurologiques* 1984; **11**: 156-9.

71. Santos-Garcia D, Suarez-Castro E, Ernandez J, et al. Predictors of Mortality in Nondemented Patients With Parkinson Disease: Motor Symptoms Versus Nonmotor Symptoms. *Journal of geriatric psychiatry and neurology* 2018; **31**: 19-26.

72. Sawada H, Oeda T, Umemura A, et al. Baseline C-Reactive Protein Levels and Life Prognosis in Parkinson Disease. *PloS one* 2015; **10**: e0134118.

73. Schneider Medeiros M, P Reddy S, P Socal M, Schumacher-Schuh AF, Mello Rieder CR. Occupational pesticide exposure and the risk of death in patients with Parkinson's disease: an observational study in southern Brazil. *Environmental health : a global access science source* 2020; **19**: 68.

74. Thaler A, Kozlovski T, Gurevich T, et al. Survival rates among Parkinson's disease patients who carry mutations in the LRRK2 and GBA genes. *Movement disorders : official journal of the Movement Disorder Society* 2018; **33**: 1656-60.

75. Velseboer DC, Broeders M, Post B, et al. Prognostic factors of motor impairment, disability, and quality of life in newly diagnosed PD. *Neurology* 2013; **80**: 627-33.

76. Wang G, Li X-J, Hu Y-S, et al. Mortality from Parkinson's disease in China: Findings from a five-year follow up study in Shanghai. *The Canadian journal of neurological sciences Le journal canadien des sciences neurologiques* 2015; **42**: 242-7.

77. Wills A-MA, Perez A, Wang J, et al. Association Between Change in Body Mass Index, Unified Parkinson's Disease Rating Scale Scores, and Survival Among Persons With Parkinson Disease: Secondary Analysis of Longitudinal Data From NINDS Exploratory Trials in Parkinson Disease Long-term Study 1. *JAMA neurology* 2016; **73**: 321-8.

78. Xie T, Liao C, Lee D, et al. Disparities in diagnosis, treatment and survival between Black and White Parkinson patients. *Parkinsonism & related disorders* 2021; **87**: 7-12.

79. Zhang Y, Wang C, Wang Y, et al. Mortality from Parkinson's disease in China: Findings from a ten-year follow up study in Shanghai. *Parkinsonism & related disorders* 2018; **55**: 75-80.

80. Kim R, Jun J-S. Impact of Overweight and Obesity on Functional and Clinical Outcomes of Early Parkinson's Disease. *Journal of the American Medical Directors Association* 2020; **21**: 697-700.

81. Levy G, Tang MX, Cote LJ, et al. Motor impairment in PD: relationship to incident dementia and age. *Neurology* 2000; **55**: 539-44.

82. Gray WK, Hildreth A, Bilclough JA, Wood BH, Baker K, Walker RW. Physical assessment as a predictor of mortality in people with Parkinson's disease: a study over 7 years. *Movement disorders : official journal of the Movement Disorder Society* 2009; **24**: 1934-40.

83. Marras C, McDermott MP, Rochon PA, et al. Survival in Parkinson disease: thirteen-year follow-up of the DATATOP cohort. *Neurology* 2005; **64**: 87-93.

84. Weintraub D, Chiang C, Kim HM, et al. Association of Antipsychotic Use With Mortality Risk in Patients With Parkinson Disease. *JAMA neurology* 2016; **73**: 535-41.

85. Zhang X, Molsberry SA, Schwarzschild MA, Ascherio A, Gao X. Association of Diet and Physical Activity With All-Cause Mortality Among Adults With Parkinson Disease. *JAMA network open* 2022; **5**: e2227738.

86. Backstrom DC, Eriksson Domellof M, Linder J, et al. Cerebrospinal Fluid Patterns and the Risk of Future Dementia in Early, Incident Parkinson Disease. *JAMA neurology* 2015; **72**: 1175-82.

87. Borda MG, Pérez-Zepeda MU, Jaramillo-Jimenez A, et al. Frailty in Parkinson's disease and its association with early dementia: A longitudinal study. *Parkinsonism Relat Disord* 2022; **99**: 51-7.

88. Camacho M, Macleod AD, Maple-Grodem J, et al. Early constipation predicts faster dementia onset in Parkinson's disease. *NPJ Parkinson's disease* 2021; **7**: 45.

89. Hiorth YH, Schulz J, Pedersen KF, Tysnes OB, Alves G. Orthostatic Hypotension and Risk of Mild Cognitive Impairment and Dementia in Parkinson's Disease. *Mov Disord Clin Pract* 2024; **11**: 1365-72.

90. Lunde KA, Chung J, Dalen I, et al. Association of glucocerebrosidase polymorphisms and mutations with dementia in incident Parkinson's disease. *Alzheimer's & Dementia* 2018; **14**: 1293-301.

91. Oftedal L, Maple-Grodem J, Dalen I, et al. Association of CSF Glucocerebrosidase Activity With the Risk of Incident Dementia in Patients With Parkinson Disease. *Neurology* 2023; **100**: e388-e95.

92. Pedersen KF, Larsen JP, Tysnes O-B, Alves G. Prognosis of mild cognitive impairment in early Parkinson disease: the Norwegian ParkWest study. *JAMA neurology* 2013; **70**: 580-6.

93. Pedersen KF, Larsen JP, Tysnes O-B, Alves G. Natural course of mild cognitive impairment in Parkinson disease: A 5-year population-based study. *Neurology* 2017; **88**: 767-74.

94. Stoker TB, Camacho M, Winder-Rhodes S, et al. Impact of GBA1 variants on long-term clinical progression and mortality in incident Parkinson's disease. *Journal of neurology, neurosurgery, and psychiatry* 2020; **91**: 695-702.

95. Szwedo AA, Dalen I, Pedersen KF, et al. GBA and APOE Impact Cognitive Decline in Parkinson's Disease: A 10-Year Population-Based Study. *Movement Disorders* 2022; **37**: 1016-27.

96. Williams-Gray CH, Goris A, Saiki M, et al. Apolipoprotein E genotype as a risk factor for susceptibility to and dementia in Parkinson's disease. *Journal of neurology* 2009; **256**: 493-8.

97. Williams-Gray CH, Evans JR, Goris A, et al. The distinct cognitive syndromes of Parkinson's disease: 5 year follow-up of the CamPaIGN cohort. *Brain* 2009; **132**: 2958-69.

98. Winder-Rhodes SE, Evans JR, Ban M, et al. Glucocerebrosidase mutations influence the natural history of Parkinson's disease in a community-based incident cohort. *Brain* 2013; **136**: 392-9.
